# Identification of novel prognostic risk signature of breast cancer based on ferroptosis-related genes

**DOI:** 10.1038/s41598-022-18044-8

**Published:** 2022-08-12

**Authors:** Nan Wang, Yuanting Gu, Lin Li, Jiangrui Chi, Xinwei Liu, Youyi Xiong, Shan Jiang, Wudi Zhang, Chaochao Zhong

**Affiliations:** 1 Department of Breast Surgery, The First Affiliated Hospital of Zhengzhou University, Zhengzhou, 450052 Henan People’s Republic of China; 2 Department of Plastic Surgery, The First Affiliated Hospital of Zhengzhou University, Zhengzhou, 450052 Henan People’s Republic of China

**Keywords:** Cancer, Computational biology and bioinformatics, Biomarkers, Risk factors

## Abstract

Ferroptosis is a type of cell regulated necrosis triggered by intracellular phospholipid peroxidation, which is more immunogenic than apoptosis. Therefore, genes controlling ferroptosis may be promising candidate biomarkers for tumor therapy. In this study, we investigate the function of genes associated with ferroptosis in breast cancer (BC) and systematically evaluate the relationship between ferroptosis-related gene expression and prognosis of BC patients from the Cancer Genome Atlas database. By using the consensus clustering method, 1203 breast cancer samples were clustered into two clearly divided subgroups based on the expression of 237 ferroptosis-related genes. Then differentially expressed analysis and least absolute shrinkage and selection operator were used to identify the prognosis-related genes. Furthermore, the genetic risk signature was constructed using the expression of prognosis-related genes. Our results showed that the genetic risk signature can identify patient subgroups with distinct prognosis in either training cohort or validation, and the genetic risk signature was associated with the tumor immune microenvironment. Finally, the Cox regression analysis indicated that our risk signature was an independent prognostic factor for BC patients and this signature was verified by the polymerase chain reaction and western blot. Within this study, we identified a novel prognostic classifier based on five ferroptosis-related genes which may provide a new reference for the treatment of BRCA patients.

## Introduction

Breast cancer (BC) has become the type of cancer with the highest incidence in the world^1^, and its incidence and mortality are still on the rise^2^. Although the continuous progress of comprehensive treatment methods, such as surgery, chemotherapy, radiotherapy, endocrine therapy, molecular targeted therapy, and immunotherapy, has reduced the mortality rate of BC in developed regions of the world, in general, about 30% of early BC patients will develop advanced BC, whose 5-year survival rate is only 20%^3^. BC has become a major public health problem in today's society, and it has seriously affected the safety and quality of life of human beings all over the world.

The term “ferroptosis” was coined in 2012 to describe a form of iron-dependent regulated cell death caused by the accumulation of lipid-based reactive oxygen species^4–6^. Ferroptosis is a regulated cell death^7^ that is related to non-apoptotic and oxidative damage. The characteristic changes of ferroptosis are the oxidation of polyunsaturated fatty acids containing phospholipids, the presence of redox iron, and the loss of lipid peroxide repair. Morphologically, it can be observed that mitochondria contract significantly; membrane density increases; and mitochondrial cristae decrease^8^. Many drugs targeting ferroptosis-related molecules have been developed, making it a promising strategy for the treatment of cancer. Although the exact pathophysiological function of ferroptosis is yet to be clearly confirmed, its role in human diseases has been confirmed, including neuro-degeneration^9–11^, ischemia–reperfusion injury^12^, and cancers^13–16^ such as BC^12–14^.

Chemotherapy is an important part of the comprehensive treatment of BC, but certain subtypes of BC are insensitive to chemotherapy drugs, or the acquired drug resistance of cancer cells tends to weaken the therapeutic effect of chemotherapy. Ferroptosis can significantly improve the efficacy of chemotherapy drugs in killing BC cells^18^, and it also plays a role in the radiotherapy of BC, in the immunotherapy of BC^19^, and in the targeted therapy of HER2-positive BC^20^, thus being an important way to treat BC^21^.

In the past two decades, with the emergence of whole-genome high-throughput platforms (such as microarrays and DNA/RNA deep sequencing), a variety of molecular biomarkers (related to etiology, prognosis, prediction, and diagnosis) that not only improve our understanding of cancer development and progression but also contribute to the early diagnosis and treatment of cancer have been found. Some previous studies have shown that some ferroptosis-related genes may be promising therapeutic targets for BC, such as *ACSL4* (Acyl-CoA synthetase long chain family member 4, Gene ID: 2182), *GPX4* (glutathione peroxidase-4, Gene ID: 2879), *SLC7A11* (solute carrier family 7 member 11, Gene ID: 23,657), and *SLC3A2* (solute carrier family 3 member 2, Gene ID: 6520)^22,23^. Some studies based on bioinformatics methods to find biomarkers related to ferroptosis have achieved some results in the diagnosis, prognosis, and treatment of tumors, such as for laryngeal squamous cell carcinoma^24^ and hepatocellular carcinoma^25^. In this study, we identified five ferroptosis-related genes that are differentially expressed in BC, including *ALB* (albumin, Gene ID: 213), *ANGPTL7* (angiopoietin like 7, Gene ID: 10218), *BLOC1S5-TXNDC5* (BLOC1S5-TXNDC5 readthrough (NMD candidate), Gene ID: 100526836), *IL6* (interleukin 6, Gene ID: 3569), and *NGB* (neuroglobin, Gene ID: 58157), and then developed a ferroptosis-related gene signature for BC patients. In addition, we studied the differences in the tumor immune microenvironment between different risk groups based on this model classification and made predictions on drug sensitivity.

## Materials and methods

### Data collection

Tumor samples used for the study were obtained from patients who underwent surgery at The First Affiliated Hospital of Zhengzhou University from December 2020 to January 2021 and had not received any prior anti-cancer treatment. Normal breast tissue samples obtained during surgery were used as control samples. Tissues were snap-frozen in liquid nitrogen until DNA/RNA isolation. The study was approved by the Ethics Committee of The First Affiliated Hospital of Zhengzhou University, and each patient's written informed consent was obtained. The patients’ age and clinical conditions such as immunohistochemistry are shown in Supplementary Table 1.

The mRNA sequencing (RNA-seq) data, survival data, and corresponding clinical and molecular information of BC patients were downloaded from the Cancer Genome Atlas (TCGA, https://portal.gdc.cancer.gov) database. In this study, specimens with no survival data were eliminated. In order to ensure the accuracy of the study, we removed the samples with no survival information and incomplete expression data. The “scale” function of R software was used to normalize the original data of gene expression. The 237 ferroptosis-related genes were retrieved from the FerrDb database and are listed in Supplementary Table 2.

To assess the classification performance of the top performing set of features or RNA transcripts, the performance was evaluated on the external validation dataset with accession GSE7390 obtained from the Gene Expression Omnibus (GEO) database. In order to validate the model on the external validation dataset, the external validation dataset was normalized as the training set.

### Cell lines

MDA-MB-231, MCF-10A and MCF-7 cell lines were obtained from ATCC (Shanghai, China) and cultured in DMEM (Invitrogen, Carlsbad, CA, USA) at 37˚C under 5% CO_2._

### Identification of BC subtypes related to ferroptosis

Based on the expression of ferroptosis-related genes, we used unsupervised consensus clustering to identify BC subtypes related to ferroptosis. Cluster analysis was performed using the unsupervised machine learning algorithm K-Means clustering^26^ by the “ConsensusClusterPlus” package in R (http://www.R-project.org/). The following parameters were used: clustering method based on k-means, 1,000 iterations, and 80% of tumor samples sampled in each iteration. The best cluster number “k” was determined by the relative changes in the area under the cumulative distribution function (CDF) curve, the proportion of the fuzzy clustering^27^ algorithm, and consensus matrix heatmaps. We used the median absolute deviation (MAD) method to explore and eliminate low-quality samples according to the expression of ferroptosis-related genes. To eliminate genes with low variability across patients, we kept genes with median absolute deviation higher than 0.5. After that, Cox analysis was performed using the “survival” package in R (http://CRAN.R-project.org/package=survival) to evaluate the correlation between all candidate genes and overall survival (OS) rate.

### Identification of differentially expressed genes in different BC subtypes distinguished by ferroptosis

The “limma” package was utilized to identify differentially expressed genes (DEGs) among different ferroptosis-related BC subtypes to further explore the potential molecular mechanisms of the subtypes. The LASSO regression model of DEGs was constructed, and genes with coefficient unequal to 0 were identified as prognosis-related genes.

### Construction of ferroptosis-related risk score signature

To verify the reliability of the ferroptosis-related markers that we identified with prognostic value, the risk score for each patient was calculated according to the linear combination of expression values weighted by the coefficient from the multivariate Cox regression analysis:

$$ Risk \, Score = \sum \beta_{i} *G_{i} , $$

where *β*_*i*_ is the multivariate Cox regression coefficient of the *i*th ferroptosis-related gene marker in the training set and *G*_*i*_ is the expression of the *i*th ferroptosis-related gene marker in each sample. Based on the risk scores, the samples were divided into high- and low-risk groups using the median value of risk score. The “survival” (https://cran.r-project.org/web/packages/survival/index.html) package was used for computing survival curves and for plotting the Kaplan–Meier estimator of patients in the high- and low-risk groups. The “survival ROC” package was used to construct time-dependent receiver operating characteristic (ROC) curves and to calculate the area under the ROC curve^28^ at 1-, 2-, and 3-year OS rates.

### Functional and pathway enrichment analysis of the ferroptosis-related gene markers

To comprehensively analyze the basic functions and participating pathways of the five genes used to build the risk model, gene ontology (GO) term function annotation and Kyoto Encyclopedia of Genes and Genomes (KEGG) pathway enrichment analysis^29–31^were employed with the “ClusterProfiler” package in R software^32^.

### Validation of the risk signature in external dataset

To validate the prognostic value of the risk signature, an external validation dataset (GSE7390) with BC was downloaded from GEO (https://www.ncbi.nlm.nih.gov/geo/) database. Using the risk scoring system, risk score was calculated for each patient in the external validation. The patients in the validation were then classified into high-risk and low-risk, and the log-rank test was used to evaluate the difference in OS between high-risk and low-risk groups.

### Correlation assessment between prognostic models and immune infiltrating cells

To analyze the relationship between the risk score and immune-cell characteristics, we used the CIBERSORT estimate (https://cibersort.stanford.edu/) software to quantify the immune cell fractions for the BC samples from TCGA. We evaluated the differences of the immune infiltration between high- and low-risk groups in TCGA BC dataset. Then, Pearson correlation analysis was used to analyze the correlation between immune cell infiltration and ferroptosis-related gene markers expression and risk score.

### Prediction of drug response using risk signature

To determine the relationship between ferroptosis-related genes and BC response rate to treatment drugs, we used risk scores to test the correlation between the prognostic model and the drug response rate. We analyzed the drug response of high- and low-risk groups and different drug response (CR: complete response, PR: partial response, PD: progressive disease, SD: stable disease) status samples. In addition, we drew the ROC curve of the risk score for the prediction of drug response and calculated the area under the ROC curve to further evaluate the application of risk scores in the prediction of drug response.

### Quantitative real-time PCR

Total RNA was isolated from 10 paired tissues using TriQuick Reagent (Solarbio, Shanghai, China) according to the manufacturer’s instructions. Then, the concentration and purity of the RNA solution was quantified using a NanoDrop 2000 nucleic acid protein quantifier (Thermo Fisher Scientific, Waltham, MA, USA). qRT-PCR was performed as described previously^33^. Before qRT-PCR, the extracted RNA was reverse transcribed into cDNA using the FastQuant RT kit with gDNA eraser (Tiangen, Beijing, China). The qRT-PCR reaction includes 2-µL reverse transcription product 1, 10-µL 2X SYBRGreen qPCR master mix (High ROX, Servicebio, Wuhan, China), 0.4-µL forward and reverse primers, and 7.2-µL nuclease-free water. PCR was performed in a MiniAmp thermal cycler (A37834, Thermo Fisher Scientific, Waltham, MA, USA) under the following conditions: 95 °C for 3 s followed by 40 cycles of 95 °C for 15 s and 60 °C for 30 s. RNA levels were calculated for tumor samples and paired adjacent samples using the 2-ΔCt method based on the *GAPDH* gene as an endogenous control. Primer sequences used for qRT-PCR are shown in Table 1.

**Table 1:** Primer sequences used for qRT-PCR.

Gene	Primer	Sequence(5, → 3,)	Length	Tm	GC%
ALB	Forward primer	TGCAACTCTTCGTGAAACCTATG	23	55.62	43
	Reverse primer	ACATCAACCTCTGGTCTCACC	21	55.21	52
ANGPTL7	Forward primer	GGGAACGAACACATCCACC	19	53.87	57
	Reverse primer	CAAAGTGGCTATACTCAGCGTAG	23	55.35	47
BLOC1S5-TXNDC5	Forward primer	AATGTATGAAGAATGGGATG	20	46.16	34
	Reverse primer	CCAAGGGAGATAGAGGTG	18	47.96	55
IL6	Forward primer	ACTCACCTCTTCAGAACGAATTG	23	55.04	43
	Reverse primer	CCATCTTTGGAAGGTTCAGGTTG	23	56.06	47
NGB	Forward primer	ACAGTGGGTGAGTCTCTGCT	20	56	55
	Reverse primer	CCCGTAGAGTTGGCTCCAG	19	54.87	63
GAPDH	Forward primer	CTGGGCTACACTGAGCACC	19	55.46	63
	Reverse primer	AAGTGGTCGTTGAGGGCAATG	21	57.03	52

### Western blot analysis

Western blot analysis was conducted using an SDS-PAGE electrophoresis system (Bio-Rad Laboratories, Hercules, CA, USA). Briefly, the total protein content was extracted from MCF-7, MCF-10A and MDA-MB-231 cell lines using a RIPA buffer (P0013C, Beyotime, Shanghai, China). Protein samples were separated by SDS-PAGE and transferred onto nitrocellulose membranes (88,520, Thermo Fisher Scientific, Waltham, MA, USA), which were subsequently blocked 12 h at 4 °C with 5% skimmed milk containing TBST (Tris-buffered salt solution, containing 50 mmol/L Tris–HCl, 150 mmol/L NaCl, 0.1% v/ v Tween-20, pH 7.4) solution. Antibodies against ALB (ab106582, Abcam, San Francisco, CA, USA), ANGPTL7 (orb350273, Biorbyt, Cambridge, UK), BLOC1S5-TXNDC5 (abx230909, Abbexa, Ltd, Cambridge, UK), IL6 (orb10911, Biorbyt, Cambridge, UK.), and NGB (sc-133086, Santa Cruz, Dallas, TX, USA.), and GAPDH (ab8245, Abcam, San Francisco, CA, USA.) were used as primary antibodies. The samples were incubated with horseradish peroxidase–conjugated secondary antibodies at 37 °C for 1 h. The membrane was imaged using an Amersham Imager 600 (GE Healthcare UK Limited, Little Chalfont, UK).

### Development of nomographs that integrate risk signature and sample clinical data

A prognostic nomogram based on the sample clinical data from the TCGA database and the risk signature constructed in this research was formulated in order to develop a risk model that is convenient for medical staff to individualize treatment for patients and to visualize model efficiency. The RMS package (version 5.1–4; https://cran.r-project.org/web/packages/rms/index.html) was employed to generate nomograms that included significant clinical characteristics and calibration plots.

### Statistical analysis

Samples in TCGA and GEO validation cohorts were divided into high- and low-risk groups based on the median value of risk score. Student’s *t*-test was used to compare the differences in the pathological and molecular characteristics of different groups. Univariate and multivariate Cox regression analyses were used to determine independent prognostic factors. Kaplan–Meier survival analysis and two-sided log-rank test were used to distinguish OS between stratified groups. The R software package “pROC” was used to draw ROC curves to analyze and predict the OS rate of BC patients. The Mann–Whitney test (*P* value adjusted by the BH method) was used to compare the infiltration of immune cells in the high-risk and low-risk groups. All statistical data were analyzed using the R software (version 4.0.2); *P* ≤ 0.05 was considered as threshold value for statistically significant.

### Ethics approval and consent to participate

Approval from the Ethics Committee of First Affiliated Hospital of Zhengzhou University(2020-KY-036-002). We can confirm that all of the methods were performed in accordance with the relevant guidelines and regulations.

### Informed consent

Written informed consent was obtained from the patients/participants for publication of this article. The studies involving human participants were reviewed and approved by the Ethics Committee of the First Affiliated Hospital of Zhengzhou University.

## Results

### Classification of breast cancer based on ferroptosis-related genes

**Figure 1:**
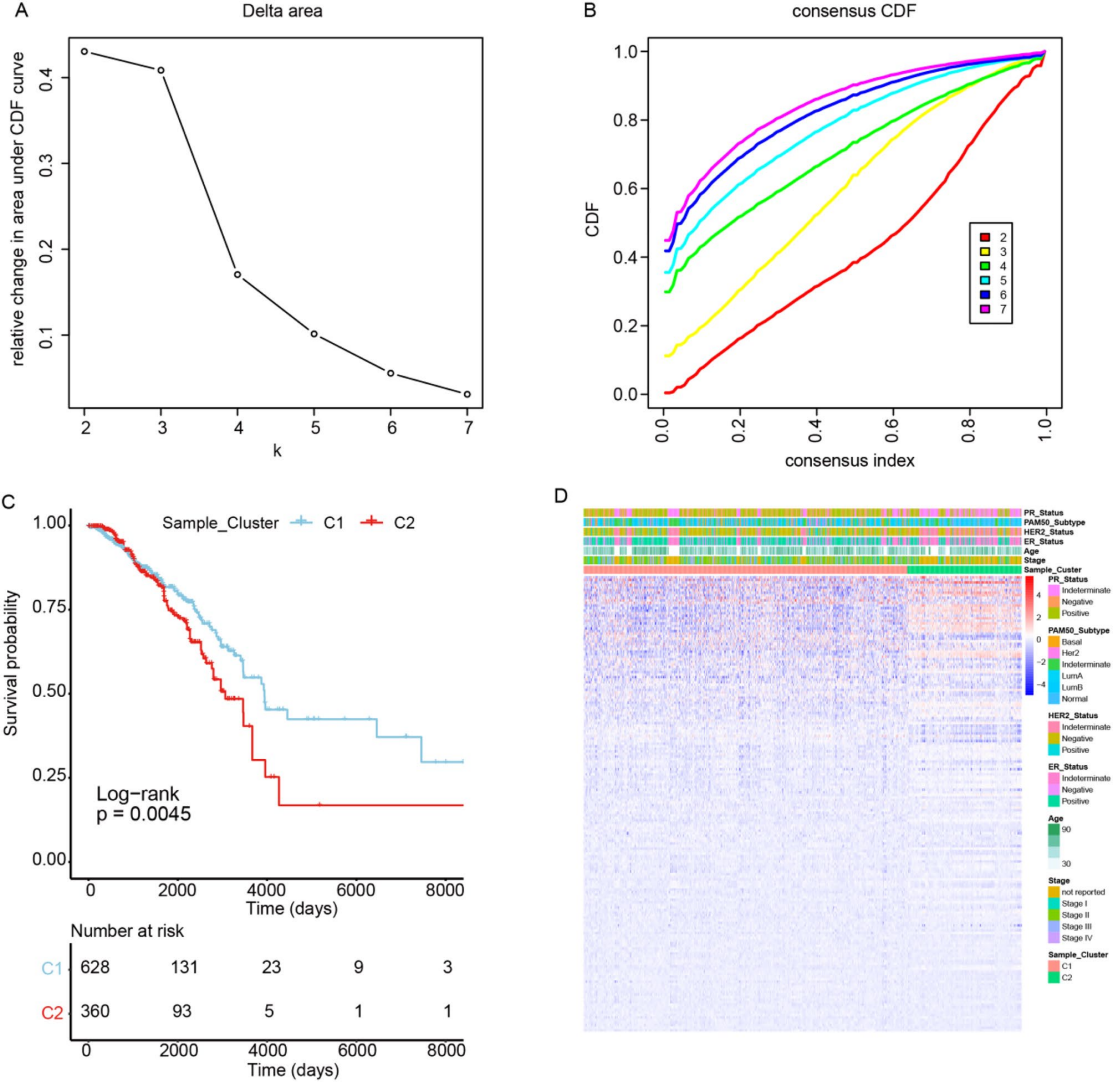
The most significant difference in the genes associated with ferroptosis appeared when breast cancer specimens were divided into two groups. (**A**) The clustering result is relatively stable when the clustering number is two. (**B**) A heat map showing clustering according to copy number. (**C**) K-M survival curves of the two clusters of BC samples. (**D**) Heatmap of differentially expressed genes in the two clusters. C1, Ferroptosis Cluster 1; C2, Ferroptosis Cluster 2.

The overall workflow of our study is displayed in the Supplementary Fig. 1. Firstly we downloaded the RNA-seq data of 1,203 BC patients from TCGA database and 237 ferroptosis-related genes from the FerrDb database, and then clustered samples according to their ferroptosis-related gene expression difference. By using the “ConsensusClusterPlus” package in R (http://www.R-project.org/), the clustering number (K) was determined by the area under the cumulative distribution function (CDF) curve, which corresponds to the largest number of clusters that induced the smallest incremental change in the area under the CDF curves. When K = 2, the clustering result was relatively stable (Fig. 1A), and the area under the CDF curves was smallest (Fig. 1B). This showed that when the BC samples were clustered into two classes (Ferroptosis Cluster 1 and Ferroptosis Cluster 2), the difference of ferroptosis-related genes in the cluster was the most obvious. At the same time, the consistent heat map with other Ks (from 3 to 7) showed that the clustering boundary was fuzzy relative to the optimal number of clusters (Supplementary Fig. 2).

To explore the survival difference between two sample clusters, we drew the K-M survival curve based on the survival data of two clusters of samples (Fig. 1C). Survival analysis showed that the patients in the cluster C1 had longer OS (log-rank, *P* = 0.0045) than that in the cluster C2. This indicates that there is indeed a difference in the survival of the two different ferroptosis-related clusters. In addition, we also found that ferroptosis-related genes were differentially expressed in the two BC sample clusters (Fig. 1D), and the ferroptosis-related genes were up-regulated expression in the cluster C2. Characteristics features of these two different groups were showed in Supplementary Table 3. There were a total of 19 genes abnormally expressed in BC among these 237 ferroptosis-related genes, including 4 up-regulated genes and 15 down-regulated genes (Table 2).

**Table 2:** 19 ferroptosis-related genes abnormally expressed in BC.

Gene name	Fold change	*P* value (wilcox test)	Down/up
TF	0.349489514	7.45E−79	Down
TFR2	2.318898382	9.53E−37	Up
CDO1	0.35278019	3.50E−99	Down
AKR1C1	0.28421946	6.56E−86	Down
AKR1C2	0.329584358	5.45E−69	Down
TP63	0.36389738	2.59E−74	Down
ENPP2	0.496832352	1.07E−106	Down
CA9	2.894057087	8.36E−09	Up
PTGS2	0.336621532	7.36E−90	Down
MT3	2.799519017	2.91E−08	Up
ALB	0.392654362	6.60E−43	Down
ANGPTL7	0.08815589	4.59E−109	Down
BLOC1S5-TXNDC5	0.407297554	1.56E−61	Down
IL6	0.301694174	9.77E−80	Down
CXCL2	0.260594915	3.41E−108	Down
IL33	0.364750266	1.03E−119	Down
HBA1	0.214120373	9.65E−38	Down
PLIN4	0.340022547	1.35E−83	Down
NGB	4.357103709	4.08E−18	Up

### Functional enrichment analysis of ferroptosis-related significantly different genes

**Figure 2:**
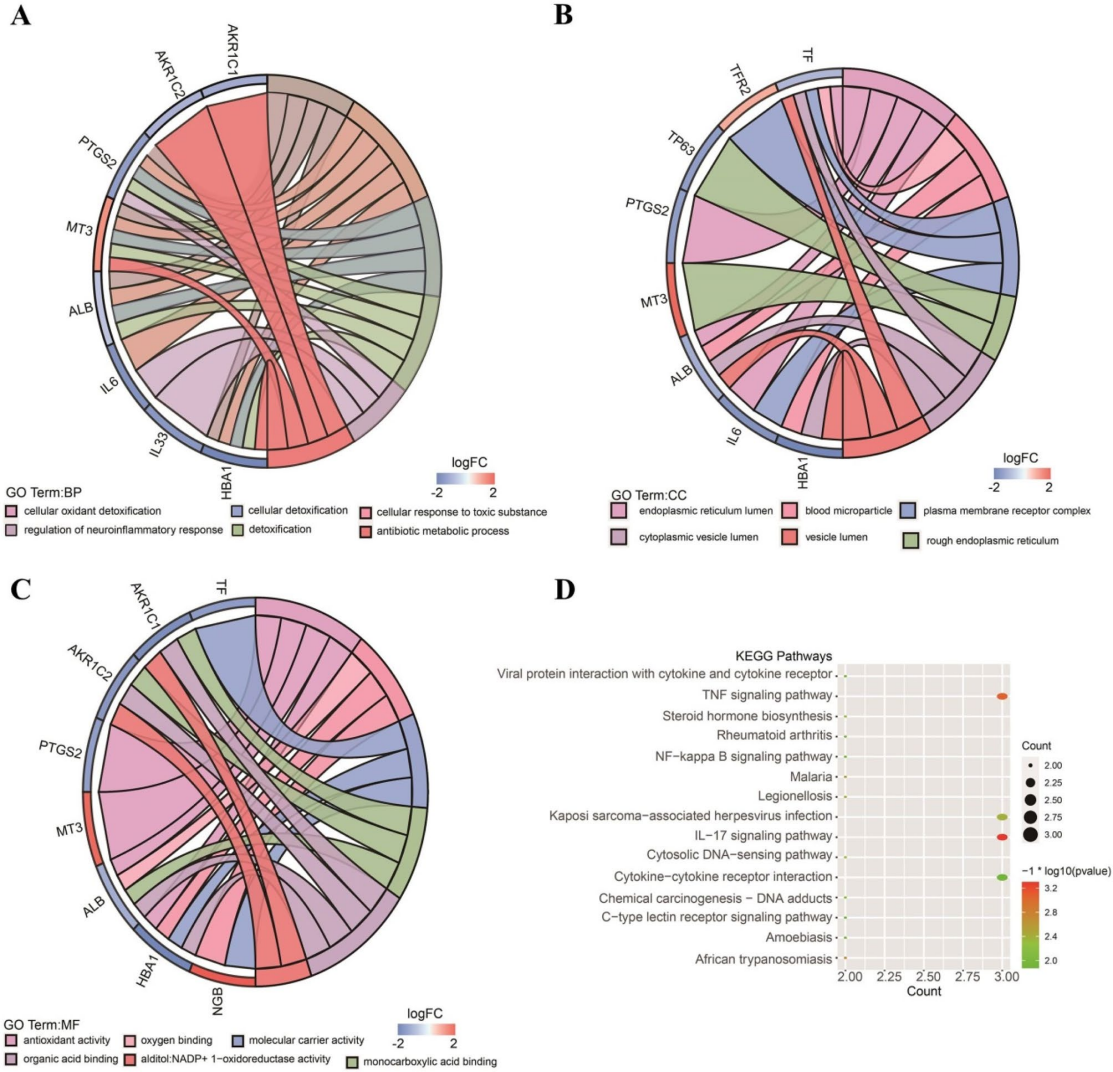
Functional enrichment analysis of the 19 ferroptosis-related SDGs. (**A**) Significantly enriched GO BP. (**B**) Significantly enriched GO CC. (**C**) Significantly enriched GO MF. (**D**) KEGG pathway enrichment analysis of the DEGs showed the top 20 enriched KEGG terms.

To elucidate the biological functions and pathways of the significantly differentially expressed genes (DEGs) in ferroptosis, the 19 genes were used in functional enrichment analysis. The results showed that 121 GO terms were enriched for biological processes (BP); six GO terms were enriched for cellular components (CC); and 43 GO terms were enriched for molecular functions^34^. Enriched BP terms included cellular oxidant detoxification (GO: 0098869), cellular response to toxic substance (GO: 0097237), cellular detoxification (GO: 1990748), and others. The GO results of the BP terms are shown in Fig. 2A, and the list of BP terms is provided in Supplementary Table 4. Previous studies indicated that the activated oxidant detoxification underlies the protective mechanism of dedifferentiated transition and lineage propagation, which affects the proliferation of cancer cells^35^. Enriched CC terms included endoplasmic reticulum lumen (GO: 0005788), blood microparticle (GO: 0072562), plasma membrane receptor complex (GO: 0098802), and others. The GO results of the CC terms are shown in Fig. 2B, and the list of CC terms is provided in Supplementary Table 5. Enriched MF terms included antioxidant activity (GO: 0016209), oxygen binding (GO: 0019825), molecular carrier activity (GO: 0140104), and others. The GO results of the MF terms are shown in Fig. 2C, and the list of MF terms is provided in Supplementary Table 6. The pathway enrichment analysis showed that twenty-seven KEGG pathways were significantly enriched (*P* < 0.05), including the TNF signaling pathway, cytosolic DNA-sensing pathway, viral protein interaction with cytokine, IL-17 signaling pathway, and others (Fig. 2D, Supplementary Table 7). Tumor necrosis factor (TNF) is a multifunctional cytokine that plays important roles in diverse cellular events such as cell survival, proliferation, differentiation, and death, which may be involved in inflammation-associated carcinogenesis^36^. As an important part of inflammation and immune system, IL-17 signaling pathway is considered to be related to the occurrence and development of tumors^37^. For example, overexpression of IL-17 from gamma delta T cells and neutrophils conspired to promote breast cancer metastasis^38^. These findings indicated that the DEGs affect the prognosis of patients by influencing the biology pathways associated with cancer.

### Construction of prognostic gene signature related to breast cancer and ferroptosis

**Figure 3:**
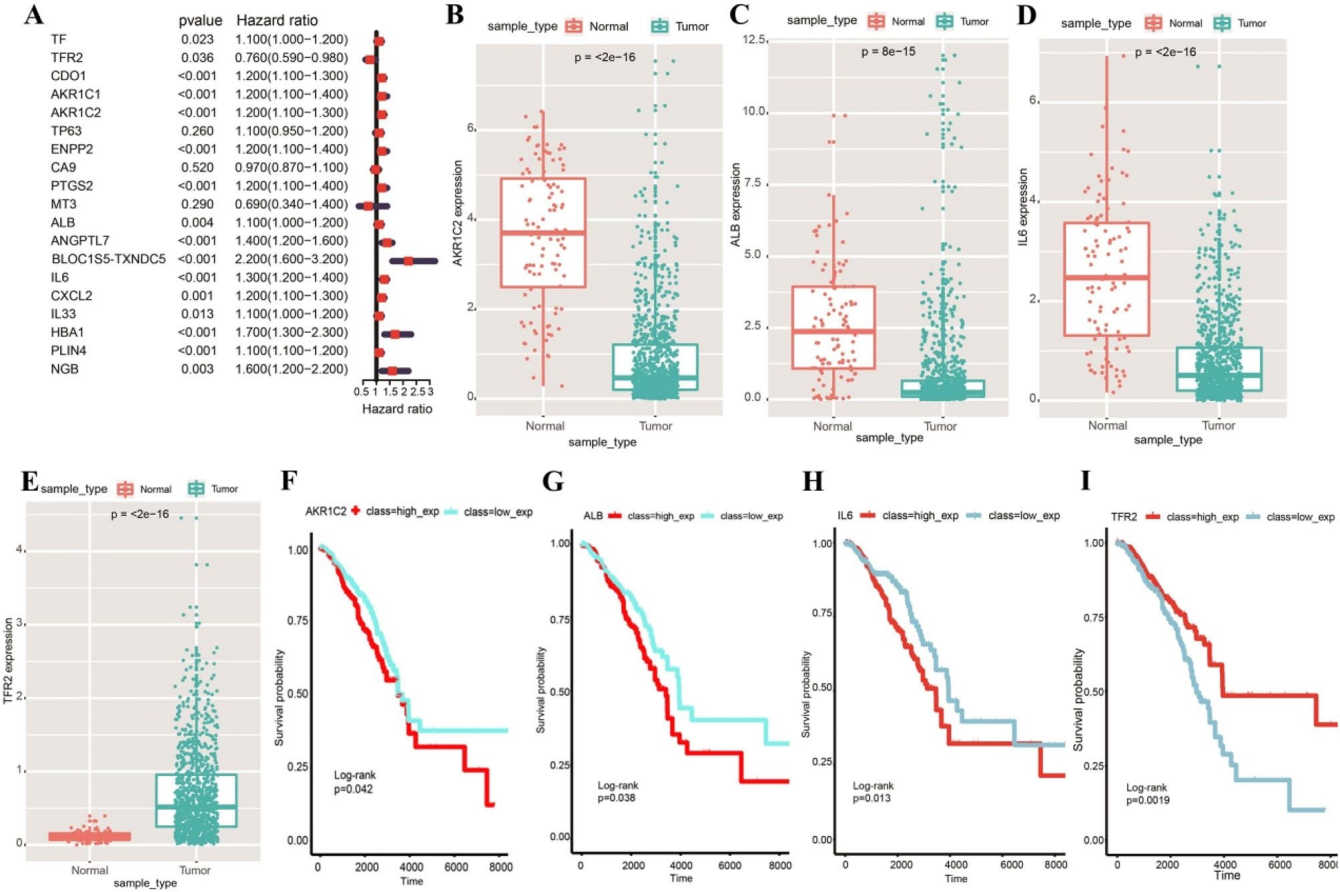
Expression of 19 ferroptosis-related DEGs in BC and their effects on the survival of BC patients. (**A**) Hazard ratio of the 19 genes by univariate Cox regression analysis. (**B**) Expression of *AKR1C2* between BC and normal samples. (**C**) Expression of *ALB* between BC and normal samples. (**D**) Expression of *IL6* between BC and normal samples. (**E**) Expression of *TFR2* between BC and normal samples. (**F**) Kaplan–Meier survival curves for patients classified into high and low *AKR1C2* expression groups (log-rank, *P* = 0.042). (**G)** Kaplan–Meier survival curves for patients classified into high and low *ALB* expression groups (log-rank, *P* = 0.038). (**H**) Kaplan–Meier survival curves for patients classified into high and low *IL6* expression groups (log-rank, *P* = 0.013). (**I)** Kaplan–Meier survival curves for patients classified into high and low *TFR2* expression groups (log-rank, *P* = 0.0019).

**Figure 4:**
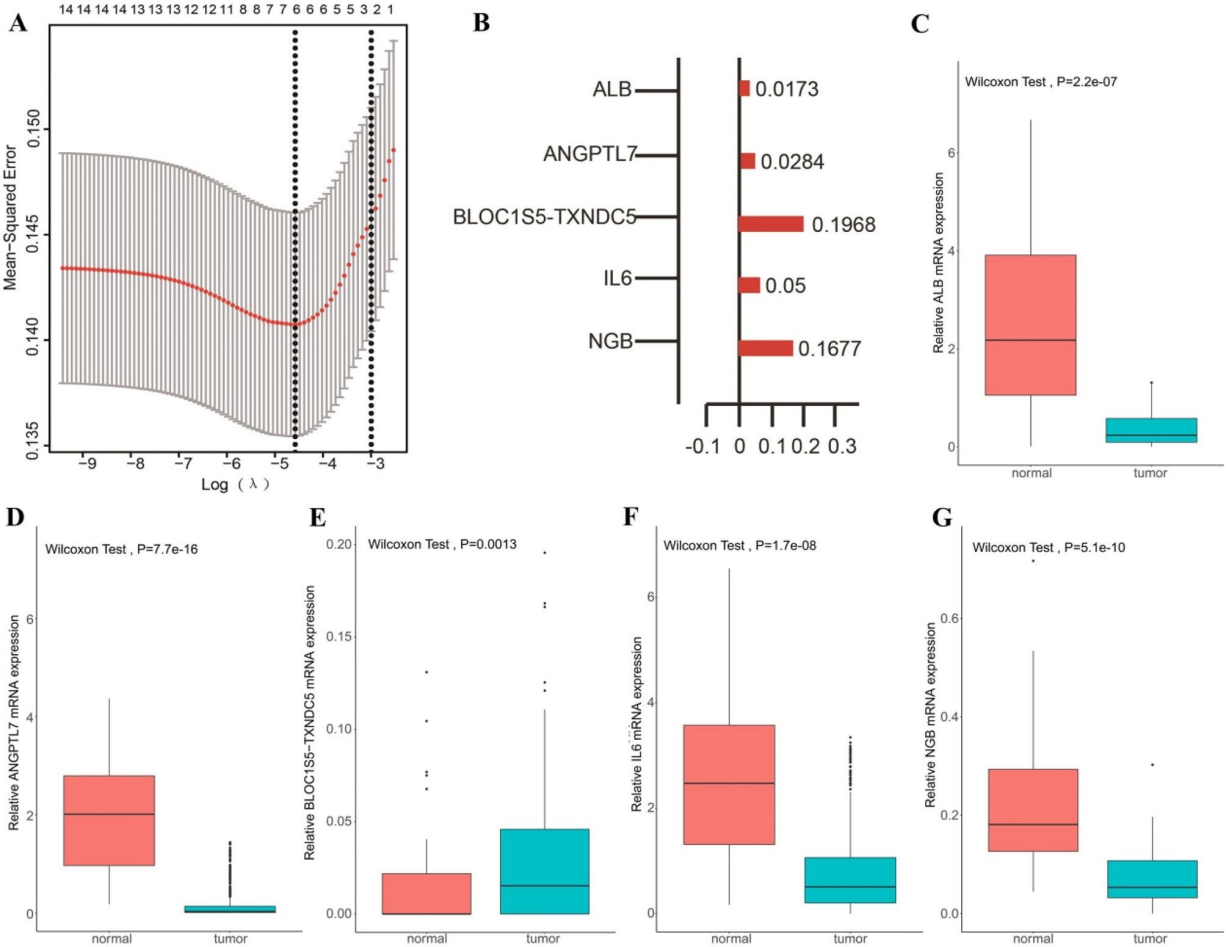
Prognostic gene signatures related to breast cancer and ferroptosis. (**A**) A LASSO regression further screened prognosis-related genes from the ferroptosis-related significantly different genes (SDGs). (**B**) LASSO Cox regression models identified five prognostic ferroptosis-related SDGs for BC. (**C**) The expression levels of *ALB* were lower in tumor issues. (**D**) The expression levels of *ANGPTL7* were lower in tumor issues. (**E**) The expression levels of *BLOC1S5-TXNDC5* were higher in tumor issues. (**F**) The expression levels of *IL6* were lower in tumor issues. (**G**) The expression levels of *NGB* were lower in tumor issues.

To further explore the prognostic potential of 19 ferroptosis-related DEGs, we performed univariate COX analysis on these 19 genes, and 16 DEGs were significant associated with patients’ prognosis (*P* < 0.05) (Fig. 3A). In order to further consider whether these 16 factors can be used as independent prognostic factors, we divided the sample group into high and low expression groups according to the median expression of each gene, and then evaluated their prognostic differences using log-rank test. Then four genes were identified as independent prognostic factors (log-rank test *P* < 0.05, including *AKR1C2*, *ALB*, *IL6*, and *TFR2*) (Fig. 3B–E). And the survival curves of other 12 genes were shown in Supplementary Fig. 8. Then, we divided the samples into high- and low-expression groups according to the median gene expression and drew the survival K-M curve (Fig. 3F–I). The results showed that there were significant differences in the expression of these genes between BC samples and normal tissues, and they had an impact on the survival of patients. The results showed that the four genes have significant prognostic value, whereas other genes do not. However, considering the limited prognostic ability of a single gene, we further explored the prognostic ability of combined multiple genes. Then, a least absolute shrinkage and selection operator (LASSO) regression analysis was performed in the 19 DEGs to identify the most robust prognostic ferroptosis-related genes combination for BC (Fig. 4A). A total of five prognosis-associated ferroptosis-related genes were included in the LASSO Cox regression model, namely *ALB*, *ANGPTL7*, *BLOC1S5-TXNDC5*, *IL6*, and *NGB* (Fig. 4B). Compared with adjacent non-tumor tissues, *BLOC1S5-TXNDC5* expression level was significantly higher in tumor tissue samples, (Fig. 4C) while the expression levels of the other four genes were significantly lower in tumor tissue samples (Fig. 4D–G). Then these five prognosis-associated ferroptosis-related genes were used to construct the prognostic signature.

### Validation of the five-gene prognostic risk signature

**Figure 5:**
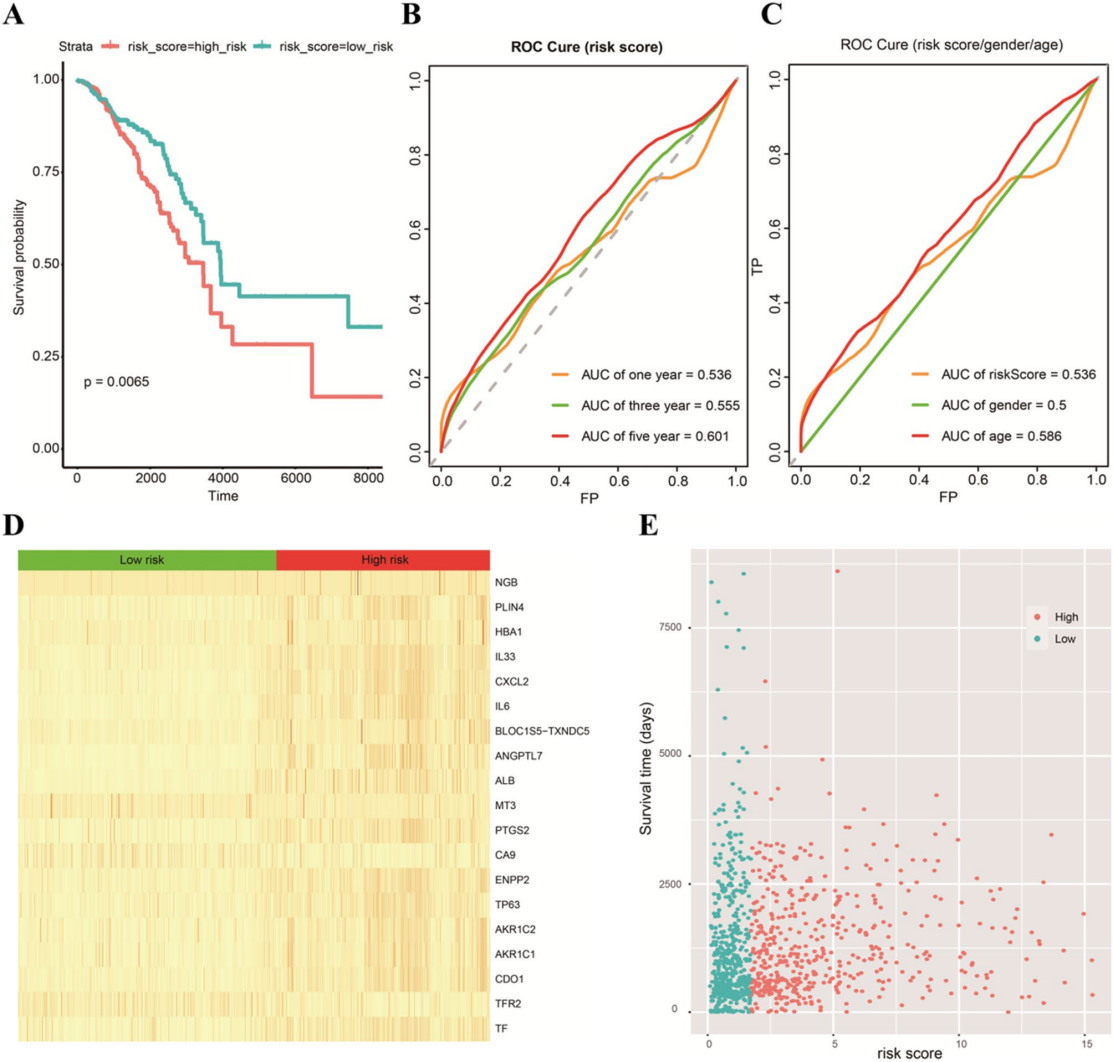
Validation of the five-gene prognostic risk score. (**A**) The survival curve shows the distinct overall survival between low- and high-risk groups. (**B**) The receiver characteristic curves of ferroptosis-related signature for predicting 1-, 3-, and 5-year disease-free survival rates. (**C**) Comparison of age and gender of patients with survival prediction. (**D**) There were differences in the expression of 19 ferroptosis-related SDGs between the high-risk group and low-risk group. (**E**) The risk score of the high-risk group and the low-risk group in our risk assessment model.

According to the median value of the prognostic risk score, patients were divided into high- and low-risk groups (each n = 494). The K-M curve indicated that the OS of higher-risk patients was significantly worse than that of lower-risk patients (Fig. 5A). As shown in Fig. 5B, AUCs for 1-, 3-, and 5-year OS rates were respectively 0.536, 0.555, and 0.601, indicating that the risk signature could predict the 5-year survival rates for the BC patients better than the 1- and 3-year survival rates. Furthermore, we performed the K-M survival analysis for BC patients with different ages (AUC = 0.586) and genders (AUC = 0.5) (Fig. 5C). In addition, we also found that there were differences in the expression of 19 differentially expressed ferroptosis-related genes in the high-risk and low-risk groups (Fig. 5D); as the risk score increased, the patients’ survival time decreased, and the death numbers increased (Fig. 5E).

### Clinical experimental validation

**Figure 6:**
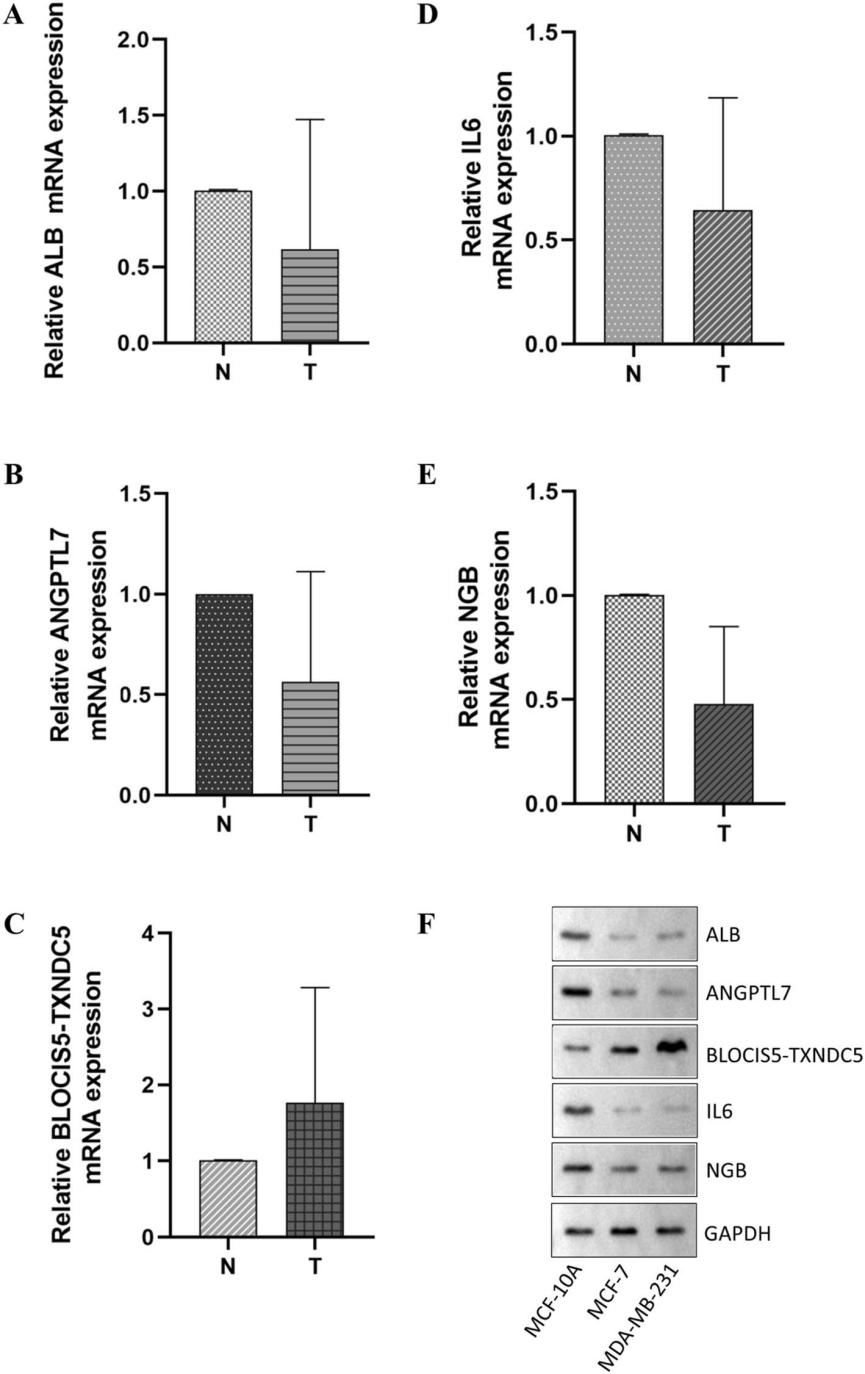
PCR and western blot analysis results of *ALB, ANGPTL7*, *BLOC1S5-TXNDC5*, *IL6*, and *NGB*. (**A**) *ALB* was down-regulated in the BC tumor tissues. (**B**) *ANGPTL7* was down-regulated in the BC tumor tissues. (**C**) *BLOC1S5-TXNDC5* was up-regulated in the BC tumor tissues. (**D**) *IL6* was down-regulated in the BC tumor tissues. (**E**) *NGB* was down-regulated in the BC tumor tissues. (**F**) Representative western blot images of ALB, ANGPTL7, BLOC1S5-TXNDC5, IL6, and NGB expressions in MCF-10A, MCF-7, and MDA-MB-231 cells.

We performed PCR validation in clinical specimens following the steps described above. We verified the five most DEGs according to the logFC values (*ALB*, *ANGPTL7*, *BLOC1S5-TXNDC5*, *IL6*, and *NGB*). The PCR results showed that *ALB*, *ANGPTL7*, *IL6*, and *NGB* were down-regulated in the BC tumor tissues, and *BLOC1S5-TXNDC5* was up-regulated in the BC tumor tissues. The details of the five genes are visualized in Fig. 6A–E (PCR experimental data is shown in Supplementary Table 8 and the relative expression of the 5 genes in PCR is shown in Supplementary Table 9). Furthermore, we performed western blot analysis of ALB, ANGPTL7, IL6, NGB and BLOC1S5-TXNDC5 protein expression levels(The uncropped original pictures were show in Supplementary Figs. 4, 5, 6, 7, 8 and 9) . We found that the protein expressions of ALB, ANGPTL7, IL6, and NGB in MDA-MB-231 and MCF-7 cell lines were lower than those in MCF-10A cell line, and the protein expression levels of BLOC1S5-TXNDC5 in MDA-MB-231 and MCF-7 cell lines were higher than those in MCF-10A cell line (Fig. 6F).

### Analysis of the relationship between risk signature and clinical characteristics

**Figure 7:**
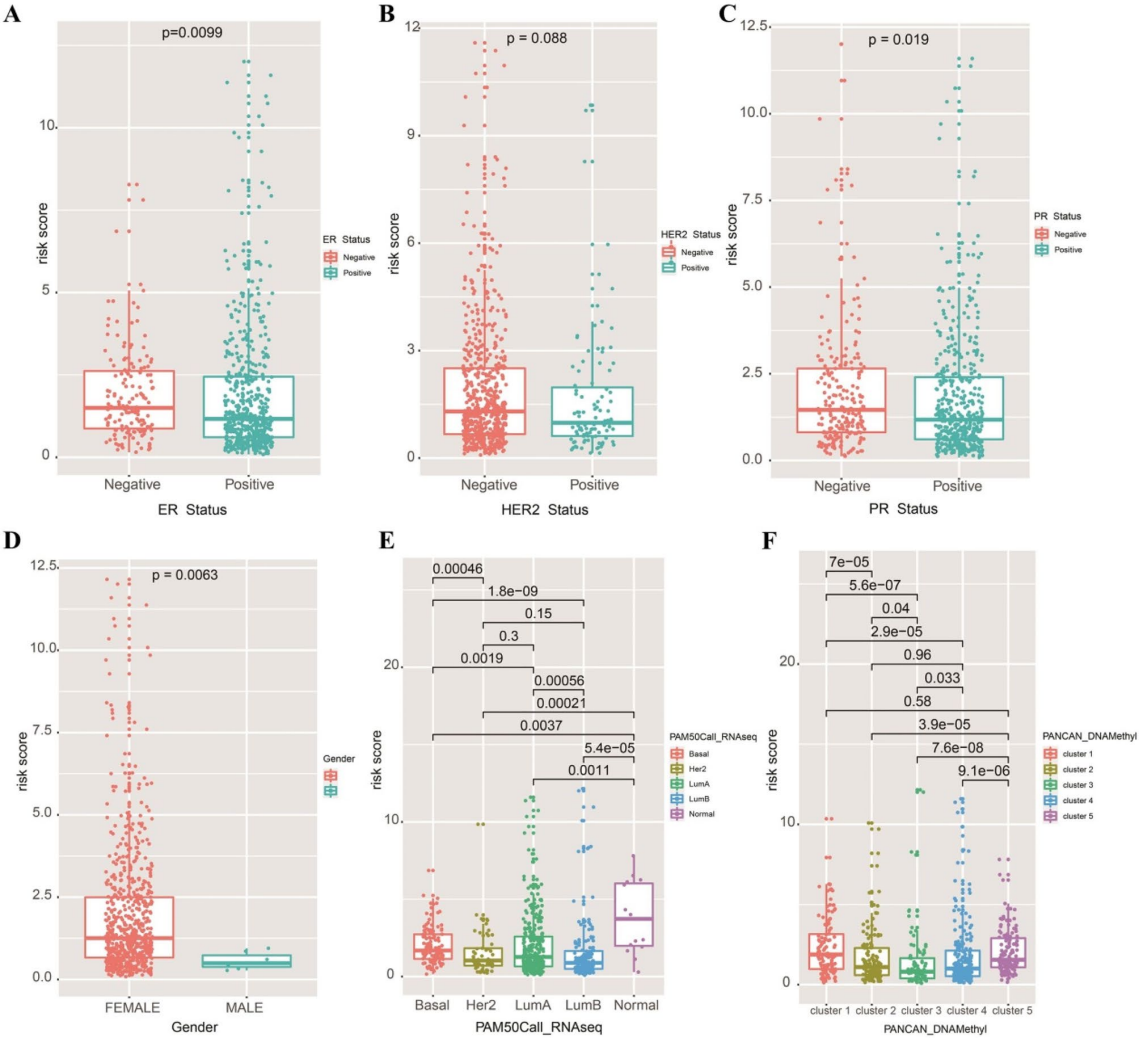
Risk analysis of BC samples with different clinical subtypes according to our risk model. (**A**) Risk assessment of BC patients with different ER status. (**B**) Risk assessment of BC patients with different HER2 status. (**C**) Risk assessment of BC patients with different PR status. (**D**) Risk assessment of BC patients with different gender. (**E**) Risk assessment of BC patients with different GEP types based on PAM50. (**F**) Risk assessment of BC patients with different methylation subtypes.

To further verify the clinical benefit of our risk score, we made a detailed analysis of BC samples with different clinical subtypes. We found that patients with positive ER, HER2, and PR status had lower risk scores than those with negative ER, HER2, and PR status (Fig. 7A–C). For patients of different genders, as shown in Fig. 7D, the risk score of male patients was lower than female patients (Wilcox test, *P* = 0.0063), indicating male patients may have better prognosis. In the analysis of BC subtypes, we found that patients with normal and basal subtypes had the highest risk signature (Fig. 7E). We also found that there were significant differences in the risk signature of patients with different methylation subtypes, the methylation subtype data of BC samples were obtained from the TCGA database, indicating that different methylation subtypes had different prognosis (Fig. 7F).

### Immune cell enrichment analysis

**Figure 8:**
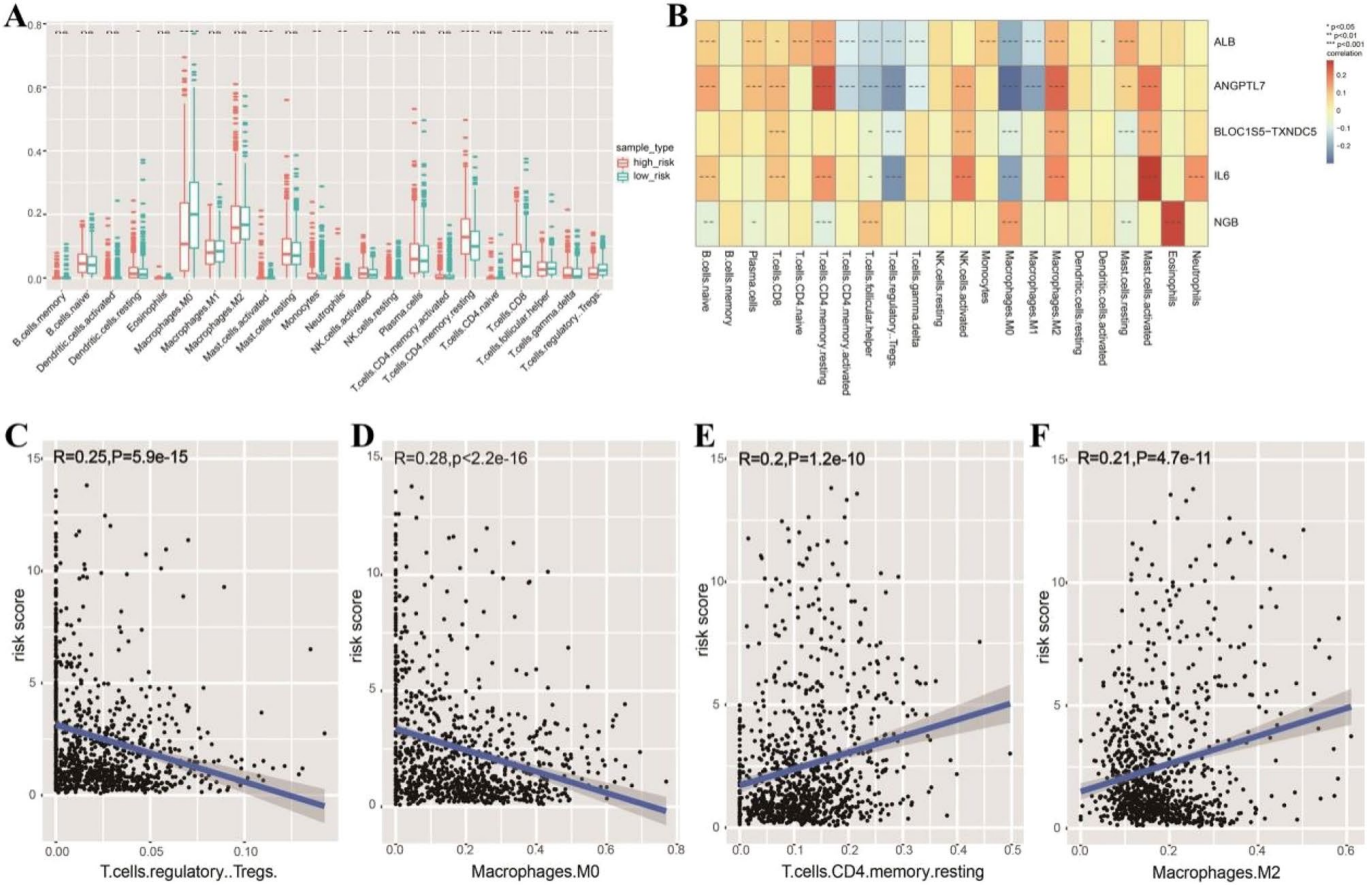
Analysis of immune infiltration in high- and low-risk groups. (**A**) There were significant differences in the infiltration of nine kinds of immune cells between the high-risk group and low-risk group. (**B**) Five ferroptosis-related prognostic markers were found to be associated with immune cell infiltration. (**C**) There was a significant negative correlation between the risk score and the infiltration degree of Tregs. (**D**) There was a significant negative correlation between the risk score and the infiltration degree of M0 macrophages. (**E**) There was a significant positive correlation between the risk score and the infiltration degree of T.cells.CD4.memory.resting. (**F**) There was a significant positive correlation between the risk score and the infiltration degree of M2 macrophages.

To further explore the relationships between the risk scores and immune cells and functions, we employed CIBERSORT to compare the differential contents of 22 immune cells between high- and low-risk groups. In terms of immune cell infiltration, significant difference was found between risk score and dendrites, macrophage, mast cells, monocytes, neutrophils, NK cells, CD8 + T-cells, CD4 + T-cells, and Treg cells (Fig. 8A). As shown in Fig. 8B, Pearson correlation analysis was used to calculate the correlation coefficient between the infiltration of 22 immune cells and the five prognosis-associated ferroptosis-related genes. The results showed that the risk signature we established were related to tumor immune infiltration, highlighting that our risk signature may influence the prognosis of patients by influencing the tumor immune microenvironment invasion. For example, the expression of *ANGPTL7* was significantly negatively correlated with the infiltration of M0 macrophages and significantly positively correlated with the degree of infiltration of CD4 + T-cells. In addition, we also performed correlation analysis between the risk assessment signature and the four immune infiltrating cells with the most significant differences in infiltration in the high- and low-risk groups. The analysis results showed that the risk score was significantly negatively correlated with the degree of infiltration of Treg cells and M0 macrophages (Fig. 8C,D), and it was significantly positively correlated with the degree of infiltration of CD4 + T-cells and M2 macrophages (Fig. 8E,F). Wang et al. found that the increasing M2 macrophage infiltration was associated with the tumor progression in esophageal squamous cell carcinoma^39^. This indicates that the infiltration of different immune cells is related to the survival of BC patients.

### Predictive performance of risk score for drug response

**Figure 9:**
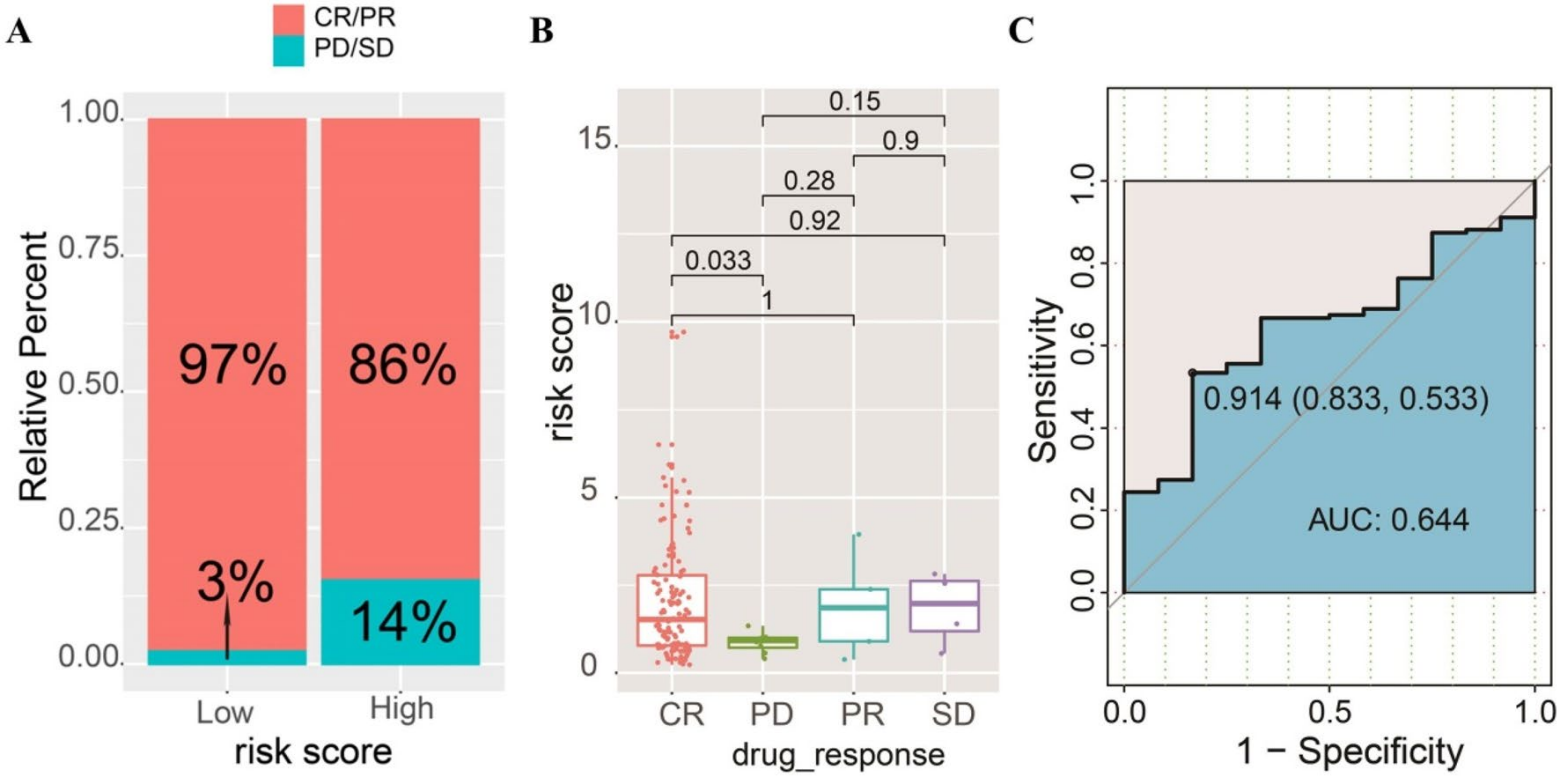
Performance of the ferroptosis-related risk score in predicting the drug resistance of tumors. (**A**) The proportion of drug response and non-response in high- and low-risk groups. (**B**) Patients in the complete response group (CR) had higher risk scores than patients with progressive disease (PD). (**C**) ROC curve showed that AUC of risk score for predicting drug response was 0.644.

To explore the performance of the ferroptosis-related risk signature, we constructed a ferroptosis-related risk signature in predicting the drug resistance of tumors, obtained clinical data of BC patients from TCGA, and analyzed the proportion of chemotherapy drug response and non-response in the high- and low-risk groups. We found that compared with patients in the high-risk group, patients in the low-risk group accounted for a higher proportion of drug responses (Fig. 9A) with better prognosis. It should be noted that the risk scores of patients in the complete response (CR) group were higher than those in the disease progression (PD) group (Fig. 9B). In clinical practice, some BCs with a very high proliferation index (i.e., highly malignant) often respond rapidly to cytotoxic drugs, thereby achieving a complete response (CR). Due to the existence of these cases, the risk score of patients in the complete response (CR) group increased. In addition, the ROC curve we drew showed that the predicted AUC of the risk score for the sample drug response was 0.644, which was higher than the random result (Fig. 9C). These analysis results indicate that the risk score we constructed has certain clinical guidance value in predicting and understanding drug response.

### Validation of the prognostic model in the GEO database and construction of the prognostic nomogram

**Figure 10:**
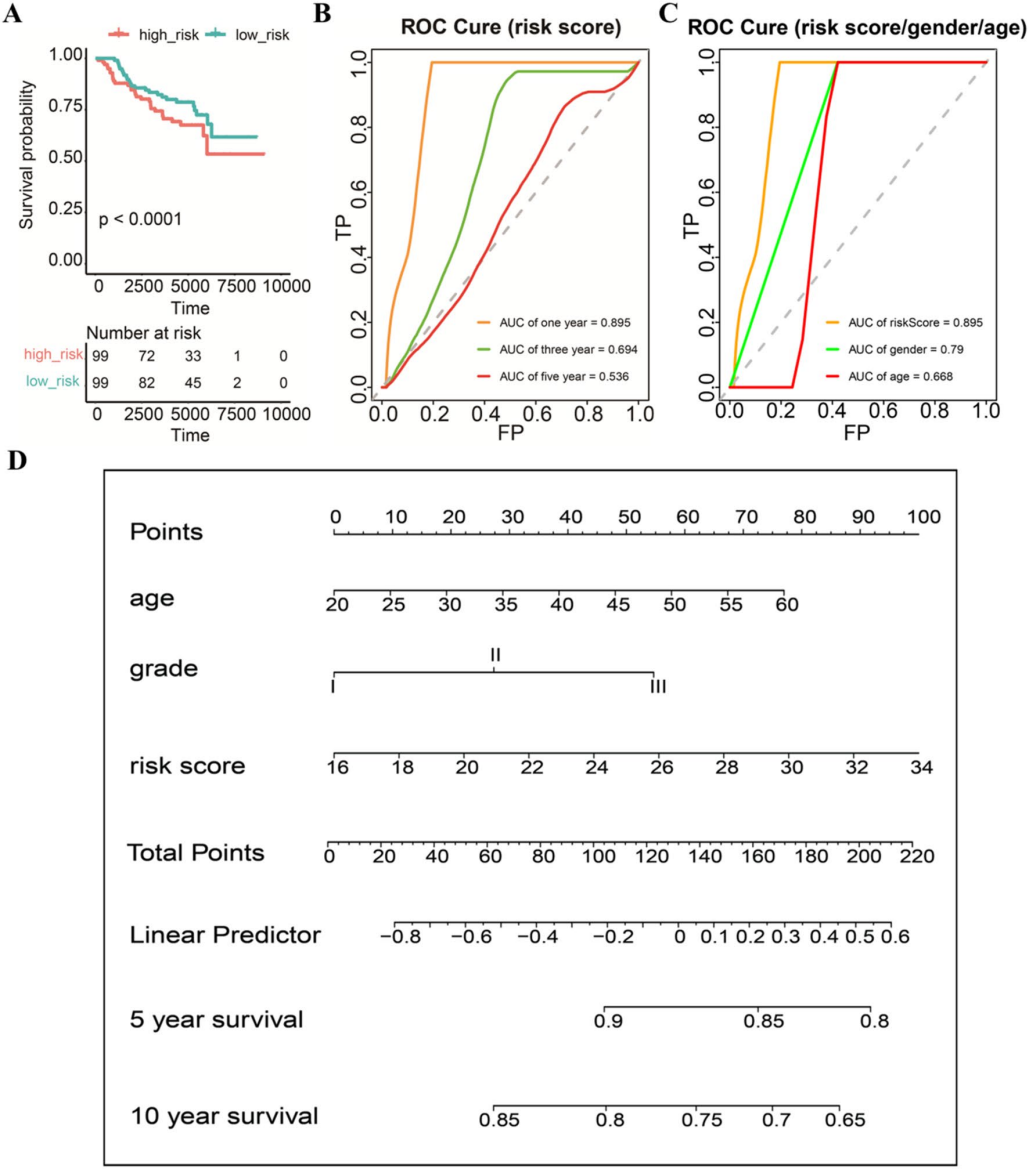
Establishment of ferroptosis-related genes-clinical nomograms for BC patients. (**A**) There was a significant difference in survival between the high-risk group and low-risk group. (**B**) The ROC curve of the validation set showed that the AUC of the risk model for 1-year survival was 0.895. (**C**) Our risk model is more effective than gender or age in predicting survival. (**D**) The nomogram for predicting overall survival of BC patients.

For verifying whether the prognostic model is robust, we applied the risk model in the GEO cohort for external validation. The patients in the GSE7390 dataset (n = 198) were divided into the high-risk group (n = 99) and low-risk group (n = 99) using the prognostic model we built. As shown in Fig. 10A, the survival probability of high-risk patients was significantly lower than that of low-risk patients (log-rank, *P* < 0.0001). As shown in Fig. 10B, the AUCs of ROCs for 1-, 3-, and 5-year OS rates were respectively 0.895, 0.694, and 0.536. Compared with gender and age as predictors, our risk model has higher predictive power (Fig. 10C). A nomogram was constructed to predict the possibility of 5-year and 10-year OS rates in BC patients by integrating the 5-FRG prognostic model and other clinicopathological characteristics (grade of the tumor and age of the patient). As shown in Fig. 10D, the nomogram demonstrated that the 5-FRG prognostic model was a valuable indicator for prognostic prediction.

## Discussion

Ferroptosis is a form of cell death completely different from apoptosis, autophagy, and necrosis^7^. It has unique morphology, gene expression, and molecular pathways. Inhibition of glutathione, GPX4 activity, and iron-dependent active oxygen burst are the key factors that induce ferroptosis^40^. Small molecule drugs have been shown to promote ferroptosis, such as elastin and RSL3^41^. Therefore, ferroptosis inducers have the potential to treat tumors^14^. Because the mechanism of ferroptosis and chemical drugs-induced apoptosis is different, iron prolapse inducers may provide a new solution to the problem of tumor drug resistance^42^.

In this study, we found for the first time that the iron sensitivity of ferroptosis-related genes can divide BC patients into two categories, and the two categories of patients show significant differences in clinical and molecular characteristics. Gene markers related to ferroptosis were established. Through LASSO regression analysis, patients were divided into high- and low-risk groups. With a focus on genetic diversity, we established a signature based on five genes: protective genes (*ALB*, *ANGPTL7*, *NGB*, and *IL6*) and risk-related genes (*BLOC1S5-TXNDC5*). Thus, BC patients were divided into high- and low-risk groups to distinguish clinical outcomes.

*BLOC1S5-TXNDC5* is also known as *MUTED-TXNDC5*, and it is an example of a conjoined gene^43^. A CG is defined as a gene that produces a transcript by combining a portion of at least one exon of each of two or more different known (parent) genes located on the same chromosome, and it is usually (95%) translated independently into different proteins. Most CGs are closely related to cancer and can be used as molecular markers for clinical diagnosis and therapeutic targets, such as *BCR-ABL* in chronic myelogenous leukemia, *ETV6-NTRK3* in secretory BC^4^, *MYB-NFIB* in adenoid cystic carcinoma^44^, and *EML4-ALK* in lung cancer^45,46^. It has been found that in addition to chromosome rearrangement, CGs can also be formed between different genes during transcription or post-transcription processing, such as trans-splicing, cis-splicing of adjacent genes (CIS-SAGE), and short homologous sequence (SHS)-mediated transcriptional sliding. A similar relationship was also observed between the expression of *BLOC1S5-TXNDC5* and the parental genes^47^. Continuous cell proliferation and altered cell cycle are both markers of cancer^46^, and studies have shown that specific or highly expressed *CIS-SAGE*^48^ in cancer cells have certain effects on cell viability and growth. With the continuous improvement of experimental techniques, CG analysis will become an important research direction in genetics and genomics.

Angiopoietin-like (ANGPTL) protein is a secreted protein, similar in structure to members of the Angiopoietin family. Some angiopoietin-like proteins have pleiotropic activity and are involved in cancer lipids, glucose energy metabolism, and angiogenesis. ANGPTL7 is a low-characteristic member of this family that is considered to be related to oxidative stress and angiogenesis. Studies have reported that ANGPTL7 plays a role in promoting vascular endothelial injury and atherosclerosis^49^, and its role in tumors has gradually attracted attention. Studies have found that *ANGPTL7* is over-expressed in colon cancer and less frequently so in ovarian cancer and expressed at a basal level in prostate cancer and lung cancer^50^. Its relationship with BC is still unclear. Neuroglobin (NGB) is a small intracellular monomeric globin that was first discovered in the neurons of the central and peripheral nervous system^51^. Over the years, a key neuroprotective effect has been attributed to the overexpression of NGB by neurons against several types of damage (such as hypoxia, oxidative stress, and hypoxia/glucose deficiency)^52–55^. In addition, recent results clearly indicate that high levels of intracellular NGB protein play a key role in BC cells’ E2, estrogen receptor alpha dependence, and antioxidant and pro-survival effects^56,57^. NGB is the key intracellular mediator of E2 in ER α + BC cells^58^, and it is a component of the BC microenvironment. NGB can be released in the tumor microenvironment by BC cells under oxidative stress conditions where it can act as an autocrine/paracrine factor to communicate cell resilience against oxidative stress and chemotherapeutic treatment^59^. Most of the samples we selected were ER + clinical specimens, and the level of *NGB* detected by PCR was consistent with the literature.

Interleukin-6 (IL6) is a pro-inflammatory cytokine released by various cells in the tumor microenvironment, including cancer cells. IL6 levels in serum and tumor sites are elevated in several cancers, including BC^60^, and are usually accompanied by poor prognosis and low survival rates in BC patients. IL6 can affect all aspects of tumorigenesis by regulating proliferation, apoptosis, metabolism, survival, angiogenesis, and metastasis^61^. IL6 can also regulate tumor treatment resistance, such as multidrug resistance^62^. The cytokine IL6 and its downstream effector STAT3 constitute a key oncogenic pathway that has been thought to be functionally connected to estrogen receptor α (ER) in BC^27^. Albumin (ALB) is one of the best-characterized markers of hepatic progenitor cells and represents a novel biomarker for this neoplasm^63^. Apart from cholangiocarcinomas (ICCs) and hepatocellular carcinomas (HCCs), hepatoid pancreatic adenocarcinoma, breast invasive ductal carcinoma, yolk sac tumor, and acinar cell carcinoma also express albumin^64^.

In short, a large number of previous studies have shown that these five genes are closely related to ferroptosis, which provides us with an important theoretical basis for constructing a risk model based on ferroptosis-related genes. Moreover, with the inclusion of some clinical and molecular features, we demonstrated that the risk score of ferroptosis-related genes is an independent prognostic indicator of OS for patients with BC. The risk score established with TCGA shows significant clinical differences between the two risk groups and can independently predict the prognosis of BC. The analysis results show that the risk score feature is a powerful prognostic indicator that can be used to classify patients and guide future treatment. After that, we built a nomogram to establish an individualized prognostic prediction model, in which the individual’s risk in the clinical environment was quantified by integrating multiple risk factors. In addition, not only did the calibration curve show a high degree of agreement between the actual survival rate and the predicted survival rate, but the prognostic-related genes we screened in the PCR detection of clinical specimens also generated results that were consistent with our predictions. The GO BP mainly comprised response to oxidative stress and cellular response to oxidative stress. The enriched KEGG pathways were ferroptosis and fatty acid biosynthesis. As observed, our study strengths include the systematic expression profile analysis, robustness of risk scoring method, and the validation across multiple platforms among multiple populations. Despite the confirmation of the predictive value of the five gene signatures in various datasets, larger-sample prospective studies are still needed to assess the clinical relevance. In addition, compared with ferroptosis, some genes in the signature may be more strongly related to other pathways in BC. In summary, our results demonstrate that the five gene markers may be potential prognostic biomarkers, providing new insight into the research and treatment of BC.

## Conclusions

Our study identified a new five-gene diagnostic signature associated with ferroptosis that can be used to predict prognosis of BC. This diagnostic signature can accurately predict the level of BC risk. It was worth noting that we had verified the reliability and applicability of this feature not only by applying it to a separate cohort but also by using PCR in detection of mRNA in our clinical tissue samples and by using western blot analysis in detection of protein in BC cell lines. In addition, the degree of invasion of immune microenvironment plays an important role in the prognosis of new genetic traits, which is helpful to find new diagnosis and treatment methods of BC.

## Supplementary Information

Supplementary Information 1.

Supplementary Information 2.

Supplementary Information 3.

Supplementary Information 4.

Supplementary Information 5.

Supplementary Information 6.

Supplementary Information 7.

Supplementary Information 8.

Supplementary Information 9.

Supplementary Information 10.

Supplementary Information 11.

Supplementary Information 12.

Supplementary Information 13.

Supplementary Information 14.

Supplementary Information 15.

Supplementary Information 16.

Supplementary Information 17.

Supplementary Information 18.
