# Supplementary figures and images for: Identification of novel prognostic risk signature of breast cancer based on ferroptosis-related genes
Source: Sci Rep. 2022 Aug 12;12:13766. doi: 10.1038/s41598-022-18044-8 (PMC9374692; doi:10.1038/s41598-022-18044-8)

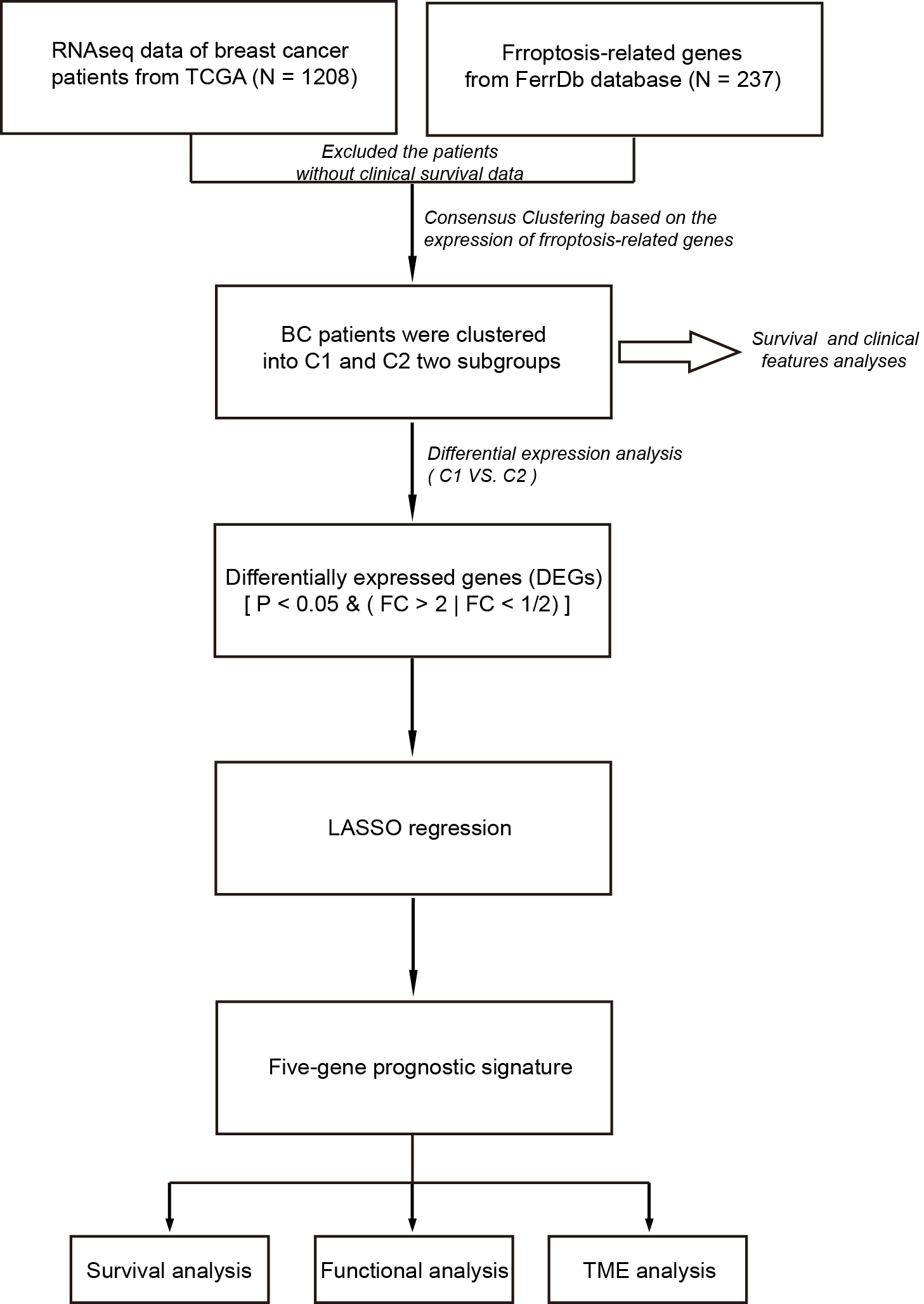

Supplement: Supplementary file 1 — Supplementary Information 1. [file 41598_2022_18044_MOESM1_ESM.tif]

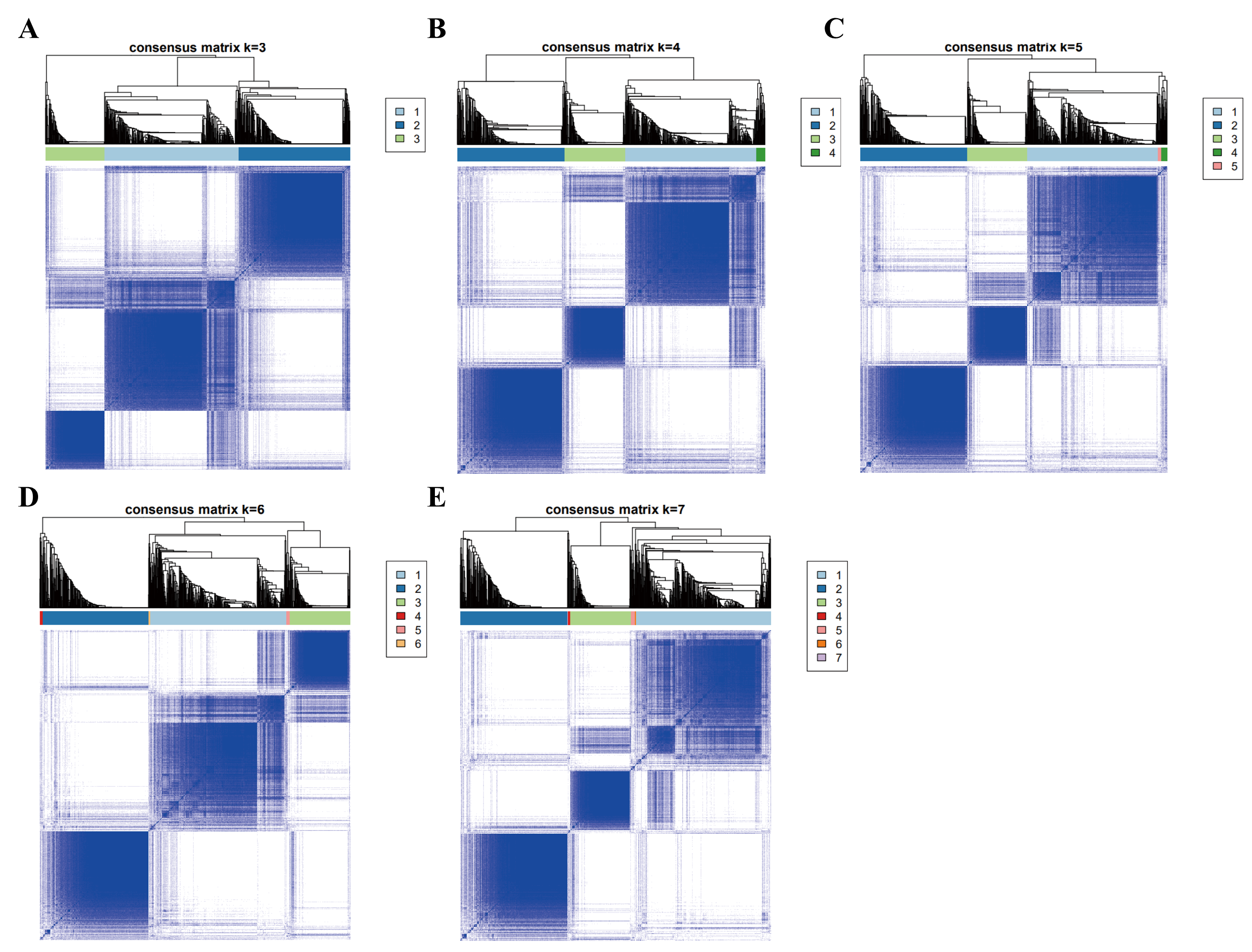

Supplement: Supplementary file 2 — Supplementary Information 2. [file 41598_2022_18044_MOESM2_ESM.tif]

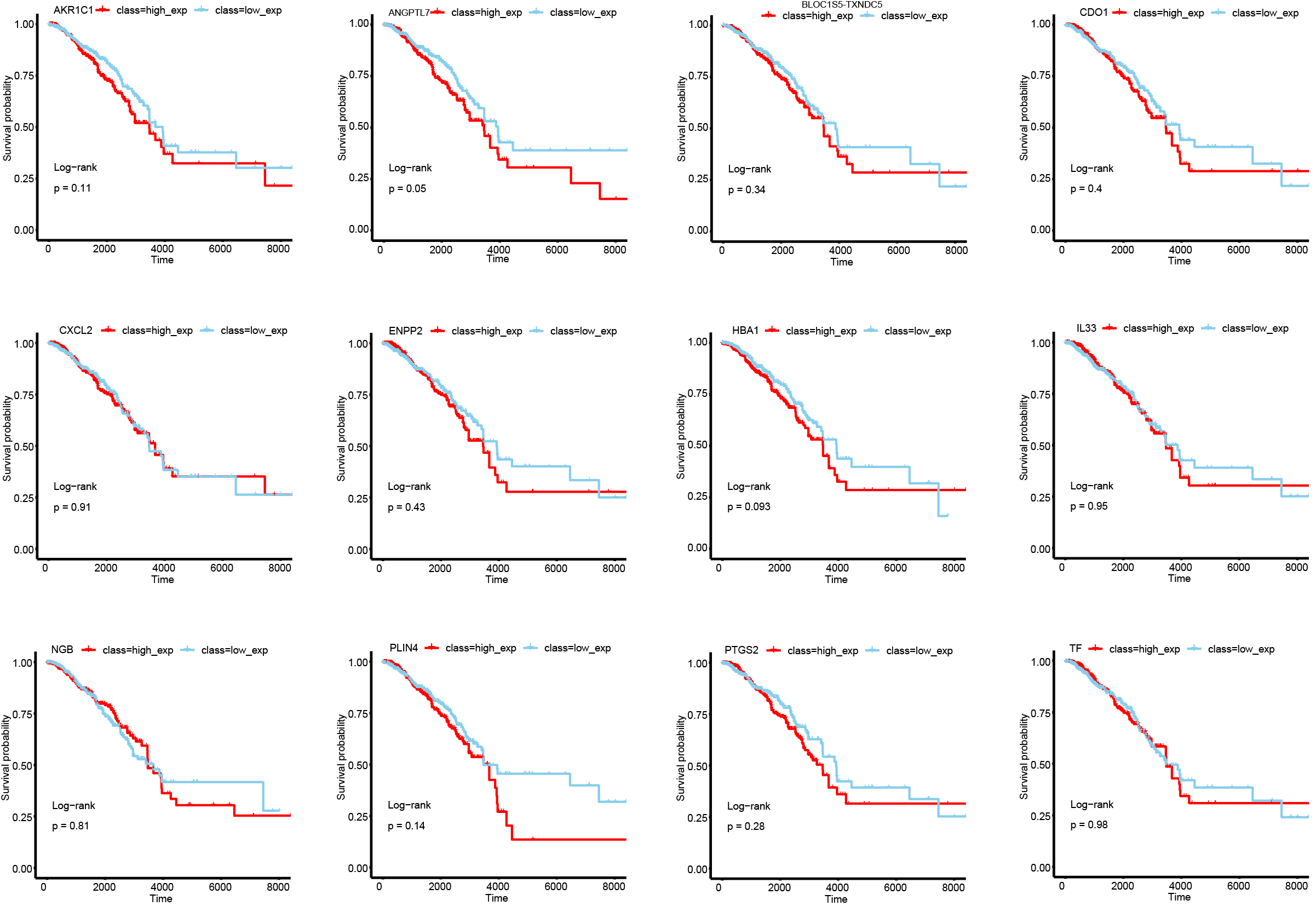

Supplement: Supplementary file 3 — Supplementary Information 3. [file 41598_2022_18044_MOESM3_ESM.tif]

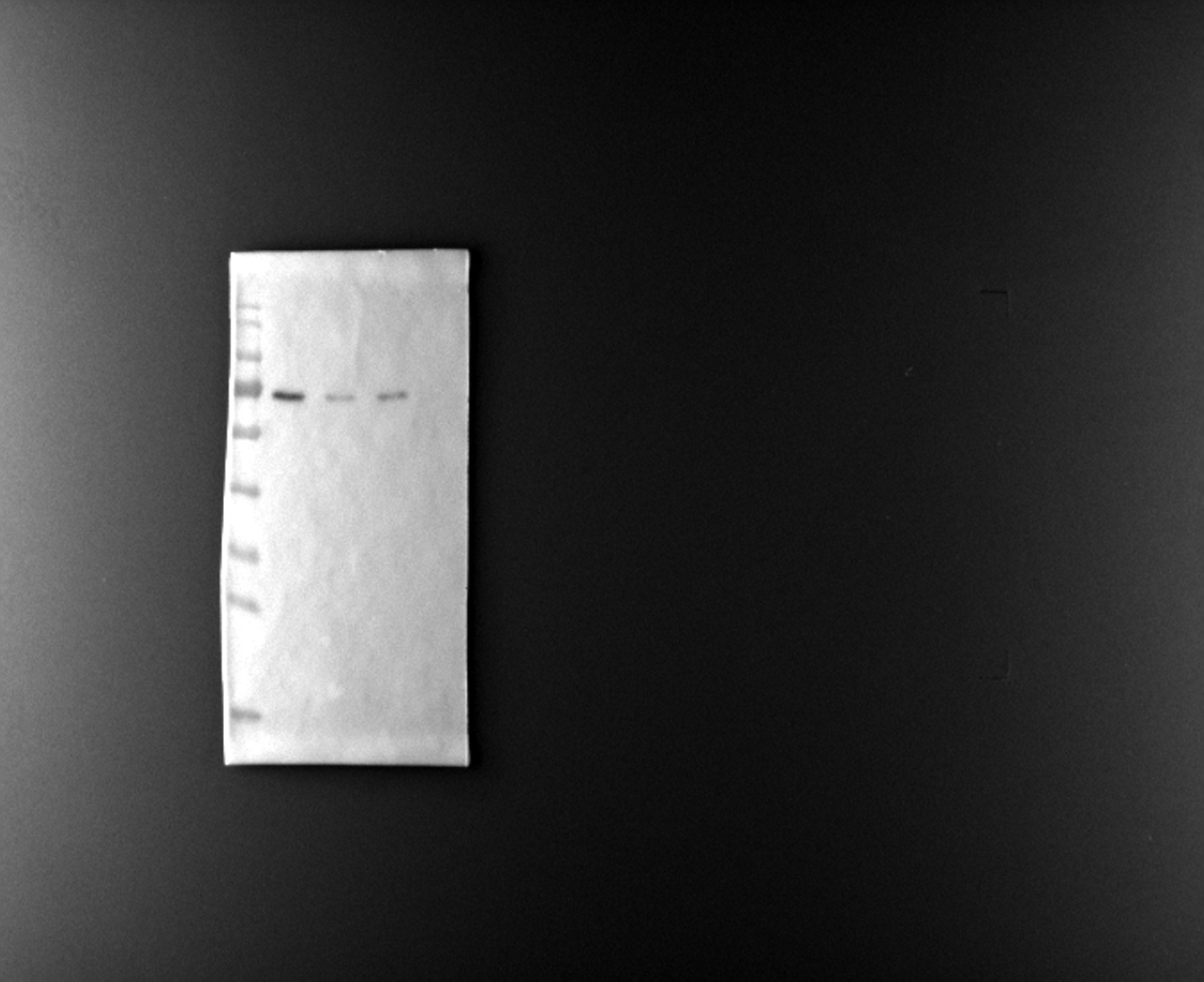

Supplement: Supplementary file 4 — Supplementary Information 4. [file 41598_2022_18044_MOESM4_ESM.tif]

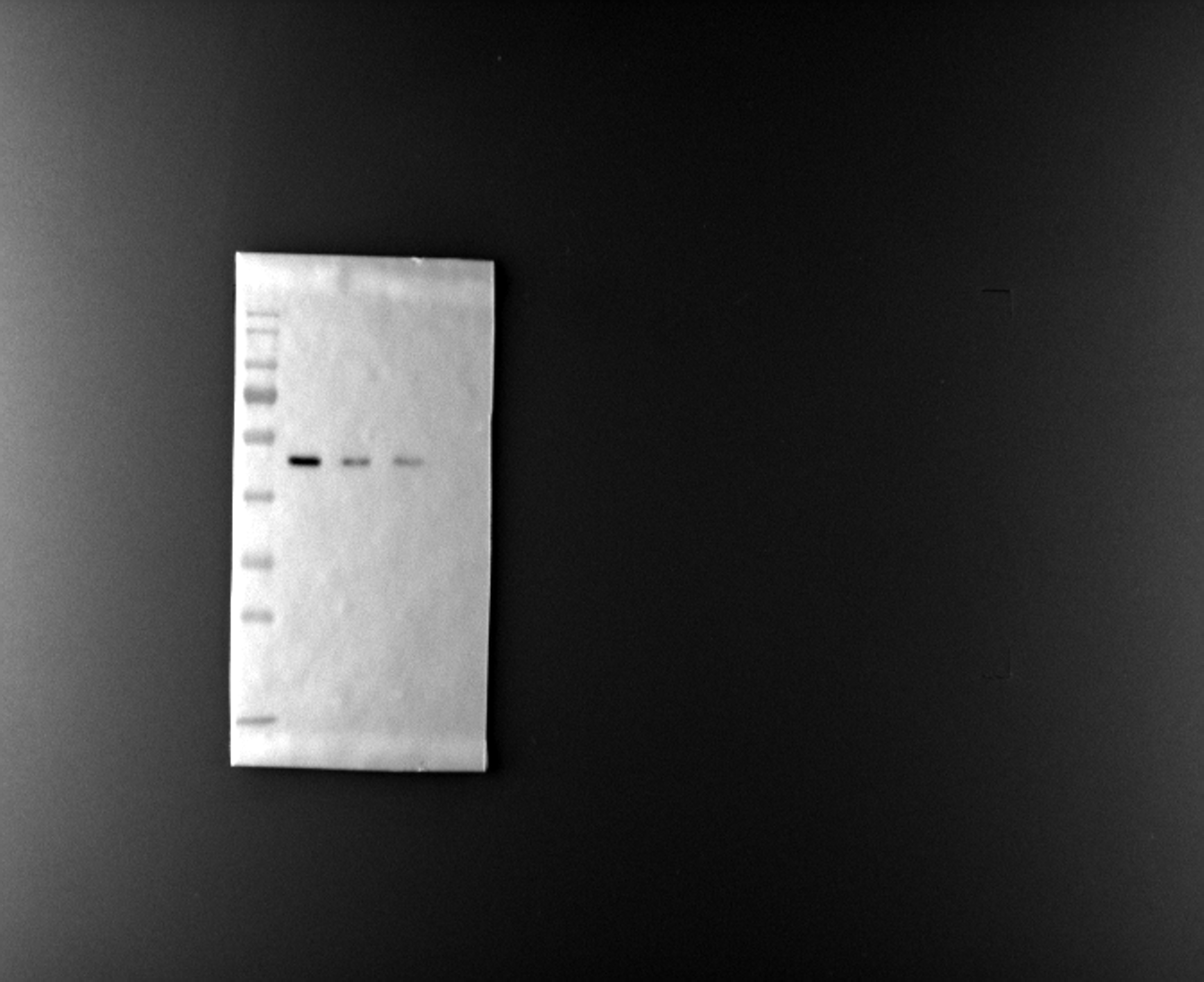

Supplement: Supplementary file 5 — Supplementary Information 5. [file 41598_2022_18044_MOESM5_ESM.tif]

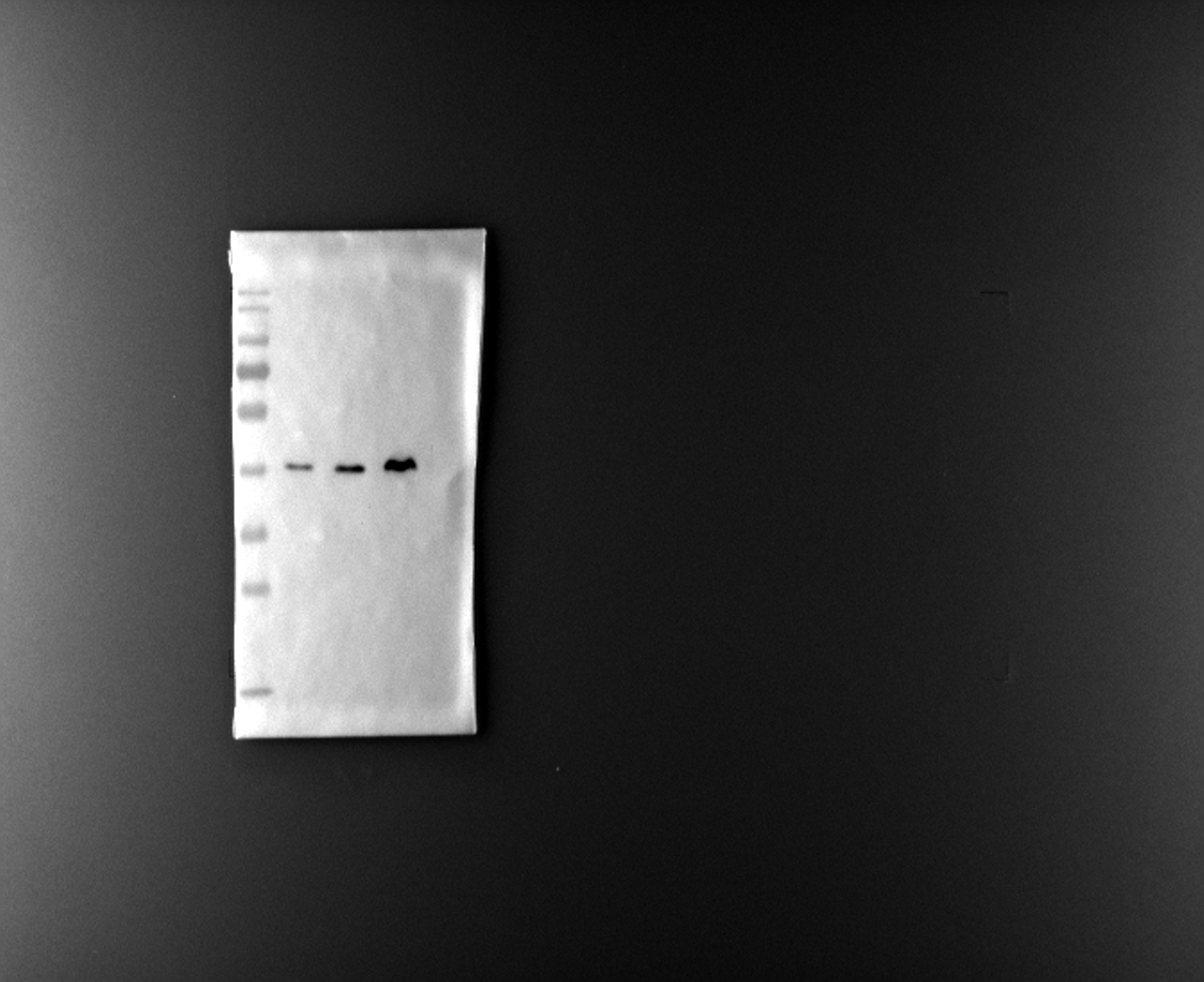

Supplement: Supplementary file 6 — Supplementary Information 6. [file 41598_2022_18044_MOESM6_ESM.tif]

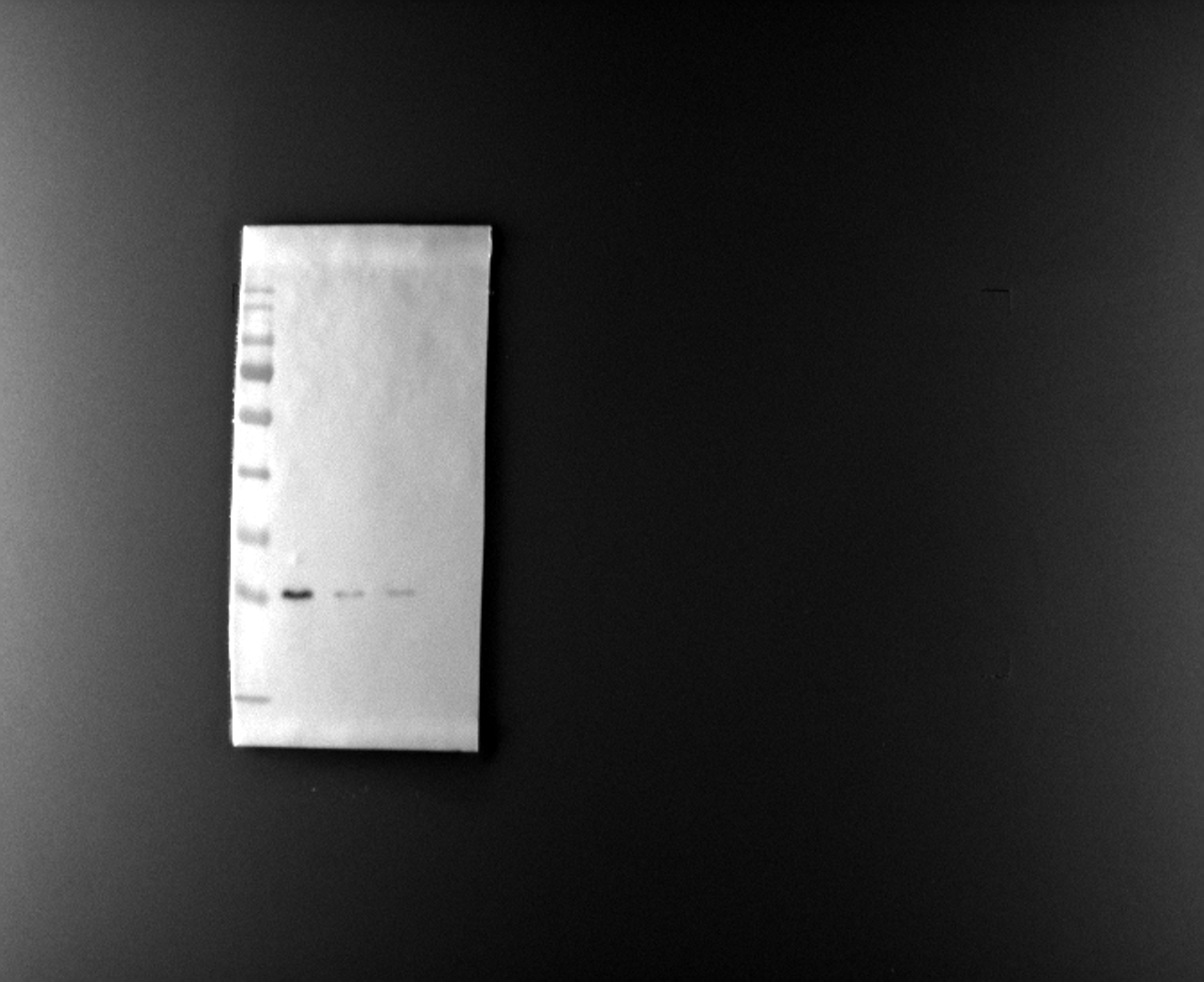

Supplement: Supplementary file 7 — Supplementary Information 7. [file 41598_2022_18044_MOESM7_ESM.tif]

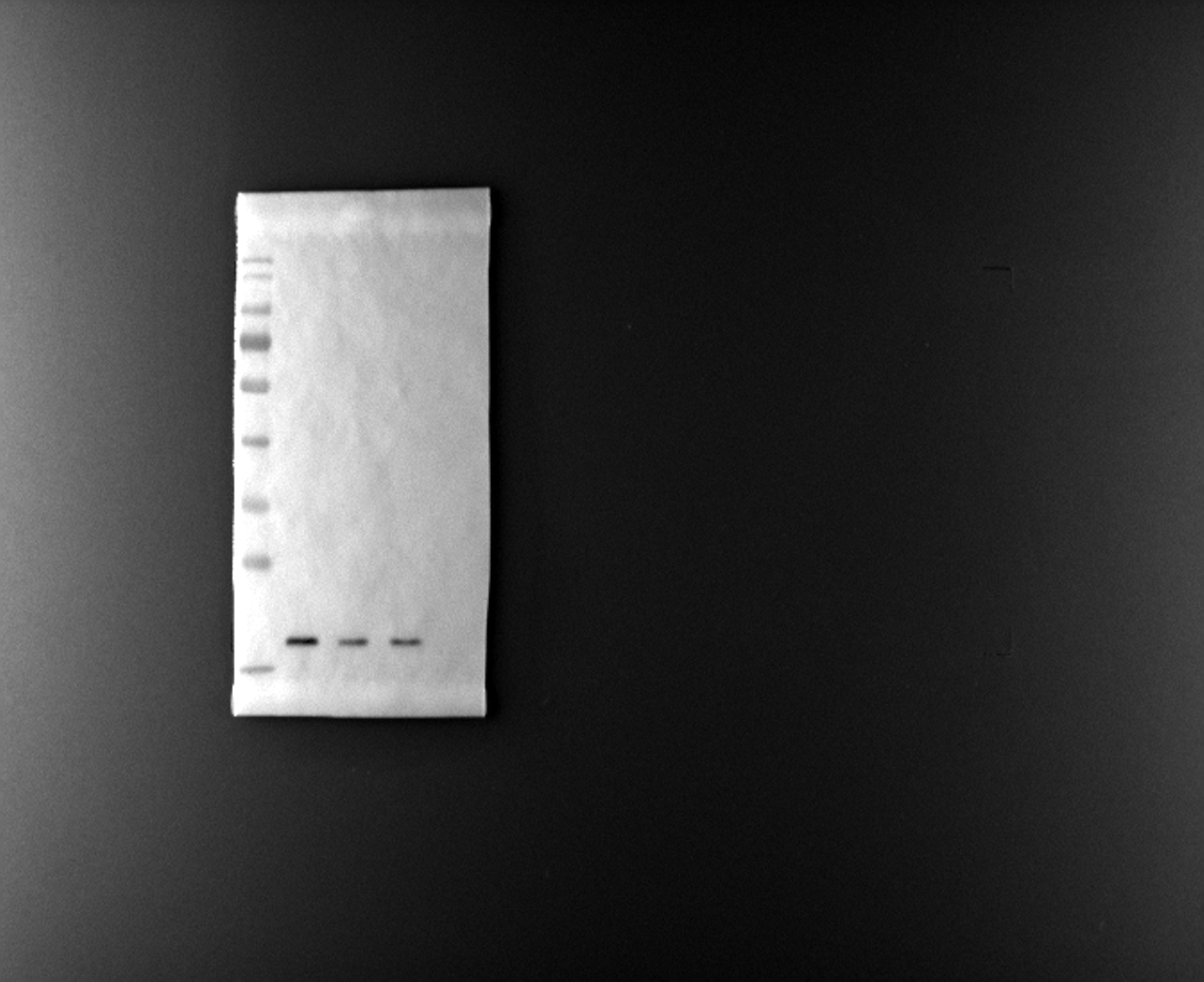

Supplement: Supplementary file 8 — Supplementary Information 8. [file 41598_2022_18044_MOESM8_ESM.tif]

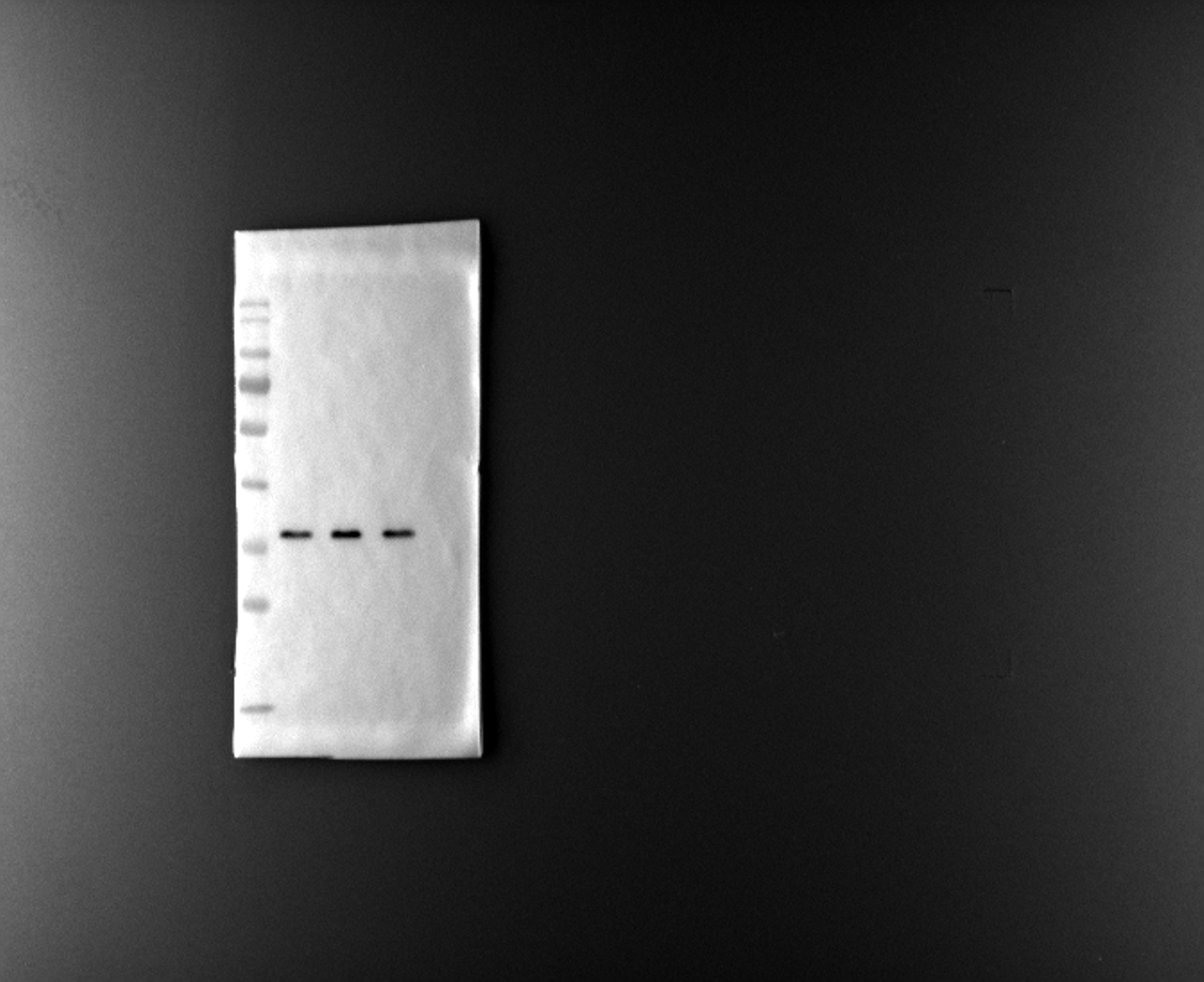

Supplement: Supplementary file 9 — Supplementary Information 9. [file 41598_2022_18044_MOESM9_ESM.tif]
